# Iron Availability Influences Protein Carbonylation in *Arabidopsis thaliana* Plants

**DOI:** 10.3390/ijms24119732

**Published:** 2023-06-04

**Authors:** Adesola J. Tola, Tagnon D. Missihoun

**Affiliations:** Groupe de Recherche en Biologie Végétale (GRBV), Department of Chemistry, Biochemistry and Physics, Université du Québec à Trois-Rivières, 3351 Boul. des Forges, Trois-Rivières, QC G9A 5H7, Canada; adesola.tola@uqtr.ca

**Keywords:** carbonylated proteins, ferritin, iron deficiency, excess iron, metal-catalyzed oxidation, α, β-unsaturated aldehydes, ketones

## Abstract

Protein carbonylation is an irreversible form of post-translational modification triggered by reactive oxygen species in animal and plant cells. It occurs either through the metal-catalyzed oxidation of Lys, Arg, Pro, and Thr side chains or the addition of α, β-unsaturated aldehydes and ketones to the side chains of Cys, Lys, and His. Recent genetic studies concerning plants pointed to an implication of protein carbonylation in gene regulation through phytohormones. However, for protein carbonylation to stand out as a signal transduction mechanism, such as phosphorylation and ubiquitination, it must be controlled in time and space by a still unknown trigger. In this study, we tested the hypothesis that the profile and extent of protein carbonylation are influenced by iron homeostasis in vivo. For this, we compared the profile and the contents of the carbonylated proteins in the *Arabidopsis thaliana* wild-type and mutant-deficient in three ferritin genes under normal and stress conditions. Additionally, we examined the proteins specifically carbonylated in wild-type seedlings exposed to iron-deficient conditions. Our results indicated that proteins were differentially carbonylated between the wild type and the triple ferritin mutant *Fer1-3-4* in the leaves, stems, and flowers under normal growth conditions. The profile of the carbonylated proteins was also different between the wild type and the ferritin triple mutant exposed to heat stress, thus pointing to the influence of iron on the carbonylation of proteins. Consistent with this, the exposure of the seedlings to iron deficiency and iron excess greatly influenced the carbonylation of certain proteins involved in intracellular signal transduction, translation, and iron deficiency response. Overall, the study underlined the importance of iron homeostasis in the occurrence of protein carbonylation in vivo.

## 1. Introduction

Protein carbonylation is a post-translational modification that occurs either due to the metal-catalyzed oxidation (MCO) of Lys, Arg, Pro, and Thr side chains or via the Michael addition of lipid peroxidation-derived α, β-unsaturated aldehydes and ketones to the side chains of Cys, Lys, and His [1,2]. Contrary to the mechanism of protein ubiquitination or phosphorylation, protein carbonylation is non-enzymatic and irreversible, but like ubiquitination, it leads to the proteasomal degradation of proteins by the proteasome system [3,4]. Protein carbonylation has been reported to play a role in the growth and development of animals and plants, oxidative stress-related diseases, and cell signalling [5,6,7,8,9]. These observations have so far been difficult to reconcile with the well-known fact that a surge in carbonylated proteins is common under stress conditions, senescence, and ageing, which then leads to cell death. Indeed, protein carbonylation has mostly been evidenced in tissues of stressed plants and in dry seeds; therefore, it is often considered to be a stress marker. However, recent genetic studies pointed to its involvement in signalling through reactive oxygen species (ROS), reactive electrophile species (RES), and phytohormones [5,6,7,8,9,10,11]. Moreover, carbonylated proteins were found to accumulate in the leaves of *Arabidopsis thaliana* in the absence of stress under normal growth conditions, thus indicating a potential role in cellular functions [12,13,14]. It remains unclear what drives protein carbonylation without oxidative stress. Whether through MCO or via the Michael addition of lipid peroxidation-derived α, β-unsaturated aldehydes and ketones, the occurrence of protein carbonylation is initiated by hydroxyl radicals (HO^•^) mostly derived from the Fenton reaction or from Haber–Weiss reactions between superoxide radicals (O_2_^•−^), H_2_O_2_, and a transition metal, such as iron (Fe) [10,15,16,17,18]. This suggests that the availability of iron may influence the occurrence of protein carbonylation in vivo.

Iron is a vital element necessary for all forms of living organisms, and iron deficiency has a profound effect on the productivity of photosynthetic organisms [19]. As a cofactor of many proteins, Fe is involved in cellular processes, including cell differentiation, respiration, and photosynthesis [20]. Many of the antioxidant enzymes and catalysts involved in electron transfer reactions require iron as a cofactor. *Arabidopsis thaliana* and other dicotyledonous species have developed an efficient strategy to acquire Fe from the soil by inducing the expression of root ferric chelate reductase encoded by FRO2 and Fe^2+^ transporter encoded by IRT1 [21,22]. In plants, cellular iron metabolism is a complex process that maintains an equilibrium between iron uptake, storage, utilization, and transport. However, there are some factors that can affect cellular iron metabolism in plants, including (1) soil pH and iron availability: soil pH can significantly affect the availability and uptake of Fe by plants because plants usually uptake iron mostly in the ferric form (Fe^3+^), but these ions have low solubility in alkaline soil [23]. Therefore, soil pH values of 5–6 are optimal for facilitating the uptake of Fe by plants. Additionally, Fe concentrations influence iron uptake because soil types with high concentrations and availability of Fe can lead to the increased uptake of Fe by the of plants [24]. (2) Iron chelators and transporters: plants have specific iron transporters, such as FRO3 and IRT1, which import iron from the soil into the root cells in non-graminaceous plants [21,22], while in grasses, iron-chelating phytosiderophores are released into the rhizosphere and form Fe^3+^-MA (mugineic acid) complexes that are taken up by specific high-affinity transporters [25]. (3) Phytohormones, such as ABA, auxin, and ethylene, can also affect iron metabolism. For example, ABA facilitates Fe-deficient responses [26] and promotes the secretion of phenolics and iron efflux from the vacuole via the upregulation of NRAMP3. Further studies reported that ABA enhances Fe translocation from the root to the shoot [26], while cytokinin and jasmonic acid inhibit Fe-deficient responses [27,28]. Overall, the regulation of cellular iron metabolism in plants is a complex process and could be influenced by a variety of biotic and abiotic factors. 

When in excess, Fe can become harmful, triggering oxidative stress via the Fenton reaction. To prevent this from happening, plants have developed some mechanisms. Such mechanisms include the use of different antioxidant systems (enzymatic and non-enzymatic antioxidants). Enzymatic antioxidants, such as superoxide dismutase (SOD), catalase (CAT), and peroxidase (POD), play a vital role in preventing the Fenton reaction by scavenging ROS and converting them to less-toxic molecules [29,30,31]. In plasmatic compartments, thiol peroxidases are more important than catalase (peroxisomes) and heme peroxidases (PODs, cell wall). They keep the hydrogen peroxide concentrations low in the cytosol, matrix, and stroma [32]. Additionally, non-enzymatic antioxidants, such as ascorbate, glutathione, and tocopherol, can directly neutralize the effect of ROS and prevent oxidative stress [33]. Plants produce some metal-chelating agents that form complexes with Fe, including phytosiderophores (Fe^3+^-mugineic acid), citrates, nicotianamine, and phylates, preventing free Fe^2+^ from triggering the Fenton reaction [25]. Moreover, plants also induce the expression of iron storage proteins, such as ferritins [34], preventing the Fenton reaction by binding Fe and sequestering it into a safe and easily accessible form [35,36]. Ferritins are conserved storage proteins that act as a dynamic “iron bank” localized within the plastids and mitochondria [37,38,39]. They play a central role in cellular iron metabolism. They can sequester large amounts of iron (up to 4500 atoms), thereby allowing iron to be released to the plant cells when needed [40,41]. For storage, ferritins transform potentially toxic ferrous (Fe^2+^) iron into a bioavailable but non-toxic ferric (Fe^3+^) ion [42]. Iron stored in ferritins cannot interact with oxygen to generate reactive oxygen species (ROS) [43]. As such, ferritins protect plants from iron-induced oxidative stress and buffer intracellular iron levels [44,45]. Four genes, *AtFer1, AtFer2, AtFer3*, *and AtFer4*, encode ferritin in *A. thaliana*. *AtFer1, AtFer3*, and *AtFer4* are expressed in vegetative and reproductive tissues, whereas *AtFer2* is only present in the seeds [38,46]. The triple-ferritin-mutant *Fer1-3-4* plants accumulate excessive iron in flowers and display a high activity of antioxidant enzymes compared to wild-type plants [47]. Considering the link between iron homeostasis and oxidative stress [47], intracellular iron could thus fuel protein carbonylation in vivo.

Previous studies proposed that iron homeostasis influences the extent of protein carbonylation in vivo, but this hypothesis has never been tested [6,48,49]. In this study, we examined this hypothesis by scrutinizing ferritin-deficient mutants and wild-type (WT) plants. Although protein carbonylation is commonly considered to be primarily caused by stress, the contents of carbonylated proteins differed between the wild-type and the ferritin-triple-mutant plants subjected to heat stress. The disruption of ferritin genes appears to alter protein carbonylation in vivo in addition to stress. In agreement with this, our results indicated that both exogenously supplied iron and endogenous iron greatly influence protein carbonylation in the plant under normal and stress conditions. Iron-deficient conditions mostly led to the carbonylation of cytosolic and plasma membrane proteins involved in the regulation of intracellular signal transduction and ribosome functions and chloroplast proteins involved in auxin and chlorophyll metabolism. 

## 2. Results

### 2.1. Iron Homeostasis Influences the Carbonylation of Proteins in A. thaliana In Vivo under Normal Growth Conditions

**Figure 1:**
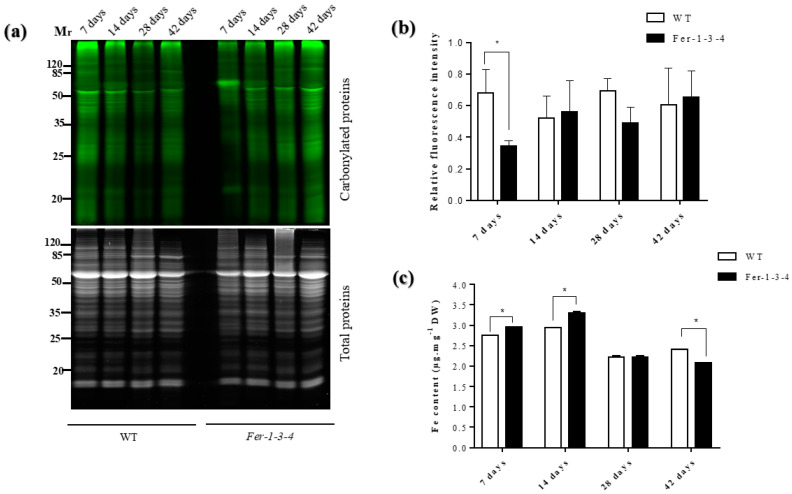
The dynamics of the protein carbonylation in the leaves of the wild-type (WT) plants and of the ferritin-mutant plants *Fer-1-3-4* over their growth period. Proteins were extracted from leaf tissues sampled from soil-grown WT and triple-ferritin-mutant *Fer-1-3-4* plants over 42 days (6 weeks) after seed stratification. (**a**) A representative gel picture of carbonylated proteins. Ten micrograms of proteins were labeled with a fluorescent hydrazide probe (Cy5.5-Hz) specific to the carbonylated proteins and then analyzed on 12.5% SDS-PAGE. *Upper gel*: Cy5.5-Hz fluorescence for the carbonylated proteins; *lower gel:* AzureRed fluorescence for the total proteins. Mr designates the protein molecular weight marker. (**b**) The abundance of carbonylated proteins based on their relative fluorescence. The quantification was based on the gel pictures shown in (**a**). The fluorescence intensity for the carbonylated proteins in each lane of the gel was normalized with the AzureRed fluorescence intensity in the same lane. The experiments were performed in biological triplicate, and the data points show the mean values ± standard error (n = 3) and an asterisk (*) indicates a significant difference between the two genotypes (multiple *t*-test with Bonferroni–Dunn correction; adjusted *p* < 0.05) (**c**) The iron content in the seedlings and leaves of adult plants. The data represent the mean ± SE (n = 3) of three independent experiments. An asterisk (*) indicates a significant difference between the two genotypes (multiple *t*-test with Bonferroni–Dunn correction; adjusted *p* < 0.05). DW denotes Dry weight. See Figure S1 in the supplementary file for replicates of Figure 1a,b.

To investigate the influence of intracellular iron on the occurrence of protein carbonylation in vivo, we compared the profiles of the carbonylated proteins between the WT and the ferritin triple mutant (*Fer-1-3-4*) of *A. thaliana* leaves at 7, 14, 28, and 42 days of plant growth, corresponding to the principal growth stage of 1.0 (early vegetative phase), stage 1.04 (leaf development), stage 3.70 (rosette growth), and stage 6.50 (flower production), respectively, after stratification [50]. Although the level of carbonylated proteins varied in both WT and the mutant depending on the growth stage (Figure 1a,b, Figure S1), the profile of the carbonylated proteins in the leaves of the mutant was similar to that of the WT, except for the samples of the first week post-stratification corresponding to the growth stage of 1.0 (early vegetative phase) [50] (Figure 1a,b, Figure S1). In these seedling samples, the levels of carbonylated proteins were lower in the mutant than in the WT. To assess the link between the intracellular iron content and the level of carbonylated proteins, the total iron content in the plant tissue was measured using a spectrophotometry-based assay that quantifies Fe^2+^-bathophenanthroline disulfonate (BPDS_3_) complexes against a set of Fe standards [51,52]. The WT and mutant seedlings (up to 14 days old) have accumulated much more iron than the adult plants, but the iron content was lower in the WT than in the mutant (Figure 1c).

**Figure 2:**
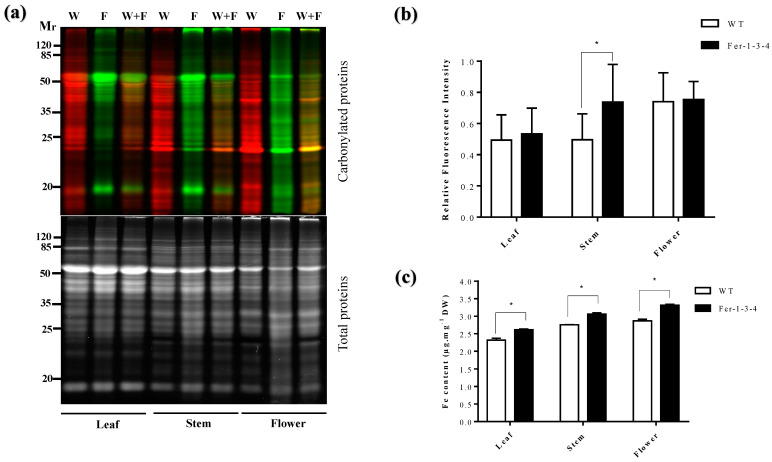
Protein carbonylation in the different organs of the wild-type (WT) plants and of the ferritin mutant plants *Fer-1-3-4*. The proteins were extracted from the leaves, stems, and flowers of six-week-old soil-grown WT and triple-ferritin-mutant *Fer-1-3-4* plants. The carbonylated proteins in the WT sample and the *Fer-1-3-4* samples were labeled with the fluorescent hydrazide probes Cy5.5-Hz (green fluorescence) and Cy7.5-Hz (red fluorescence, pseudocolor), respectively. The samples were then analyzed on 12.5% SDS-PAGE. (**a**) Each lane contains 10 µg proteins. W: WT sample; F: *Fer-1-3-4* sample; W + F: mixture of equal amounts of WT and *Fer-1-3-4* sample. *Upper gel*: Cy5.5-Hz and Cy7.5-Hz fluorescence for the carbonylated proteins; *lower gel:* AzureRed fluorescence for the total proteins. Mr designates the protein molecular weight marker. (**b**) The abundance of carbonylated proteins based on their relative fluorescence intensity. The plotted values were obtained as described in Figure 1. The experiments were performed in biological triplicate, and the data points show the mean values ± standard error (n = 3) and an asterisk (*) indicates a significant difference between the two genotypes (multiple *t*-test with Bonferroni–Dunn correction; adjusted *p* < 0.05) (**c**) The iron content in the leaves, stems, and flowers. The data represent the mean ± SE (n = 3) of three independent experiments. An asterisk (*) indicates a significant difference between the two genotypes (multiple *t*-test with Bonferroni–Dunn correction; adjusted *p* < 0.05). DW denotes Dry weight. See Figure S2 in the supplementary file for replicates of Figure 2a,b.

Since the iron pools may vary between the leaves and the other parts of the plant, we examined the profile of the carbonylated proteins in different organs of the plants. We found that the profile and the amount of the carbonylated proteins differed between the leaves, stem, and flowers and between the WT and the triple ferritin mutant *Fer-1-3-4*. There were more carbonylated proteins in the flowers than in the leaves of the WT and the mutant (Figure 2a,b, Figure S2). In addition to this, the carbonylated proteins were more abundant in the stem samples of *Fer-1-3-4* than those of the WT, and a few bands of carbonylated proteins could be distinguished between the WT and the *Fer-1-3-4* stem and flower samples. In parallel, the flowers were found to contain much more iron than the leaves and the stems of the two genotypes, and the iron content was slightly more abundant in the leaf, stem, and flower samples of the mutant than in the samples of the WT (Figure 2c). This indicated that some proteins were differentially carbonylated between the WT and the *Fer-1-3-4* mutant and between the organs depending on the iron availability.

### 2.2. Iron Homeostasis Impacts on the Occurrence of Protein Carbonylation under Stress

**Figure 3:**
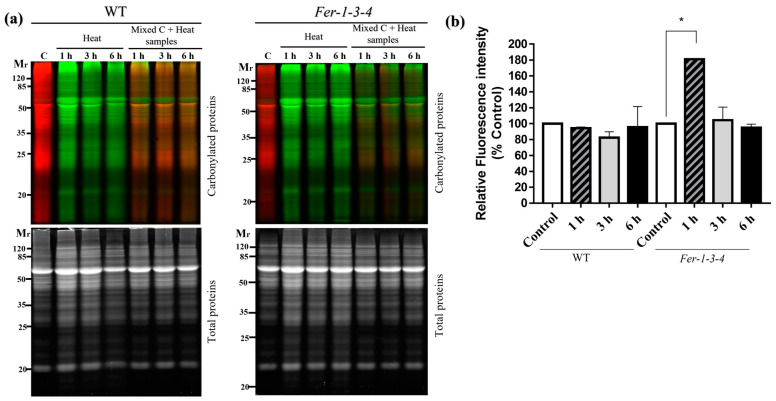
Protein carbonylation in the wild-type and *Fer-1-3-4* mutant under heat stress. The proteins were extracted from the wild-type (WT) and *Fer-1-3-4* mutant plants germinated on ^1^/_2_ MS-agar plates for 10 days. The seedlings were then exposed to a high temperature (38 °C) in the dark for 1, 3, and 6 h. The control (C) plants were kept in the growth chamber at 22 °C. After the heat treatment, the plants were returned to the growth chamber at 22 °C for 2 h before sampling. (**a**) The carbonylated proteins in the control sample and the heat-treated plant samples were labeled with fluorescent hydrazide probes Cy5.5-Hz (red fluorescence, pseudocolor) and Cy7.5-Hz (green fluorescence), respectively. The samples were then analyzed on 12.5% SDS-PAGE, either alone or mixed in equal amounts. Each lane contains 10 µg of proteins. *Upper gels*: Cy5.5-Hz and Cy7.5-Hz fluorescence for the carbonylated proteins; *lower gels:* AzureRed fluorescence for the total proteins. Mr designates the protein molecular weight marker. (**b**) The abundance of carbonylated proteins based on their relative fluorescence intensity (% of control). The plotted values were obtained as described in Figure 1. The experiments were performed in biological triplicate, and the data points show the mean values ± standard error (n = 3). An asterisk (*) indicates a significant difference between the control and heat stress treatment in the two genotypes (one-way ANOVA, followed by Tukey’s test *p* < 0.05). See Figures S3 and S4 in the supplementary file for replicates of Figure 3a.

Protein carbonylation is well known to intensify under stress conditions, but the contribution of intracellular iron is less clear. To verify whether iron availability influences protein carbonylation under stress conditions, the profiles of the carbonylated proteins were compared between the WT and the ferritin-triple-mutant *Fer-1-3-4* seedlings exposed to heat stress at 38 °C. There was much difference between the WT and the *Fer-1-3-4* as to the amount and profile of the carbonylated proteins that accumulated in response to the heat treatment. As judged by the intensity of the red and green pseudocolor fluorescence derived from the hydrazide probes used for labeling the carbonylated proteins, some fluorescent protein bands were present in the samples of the WT seedlings exposed to heat but absent in the control samples (Figure 3a,b: WT panel, lanes corresponding to *Mixed C + heat samples*, Figure S3). In contrast to the minor changes observed in the WT, the level of carbonylated proteins markedly increased in the samples of heat-treated *Fer-1-3-4* mutant compared to the samples of untreated seedlings (Figure 3: Fer-1-3-4 panel, lanes corresponding to *Mixed C + heat samples*, Figure S4). The disruption of the major ferritin gene expression in the Arabidopsis shoots thus appeared to enhance protein carbonylation in the seedlings exposed to severe heat stress and underlined the importance of iron homeostasis in the occurrence of protein carbonylation in vivo. 

### 2.3. Iron Excess and Iron-deficient Conditions Influence Protein Carbonylation in the A. thaliana Seedlings

**Figure 4:**
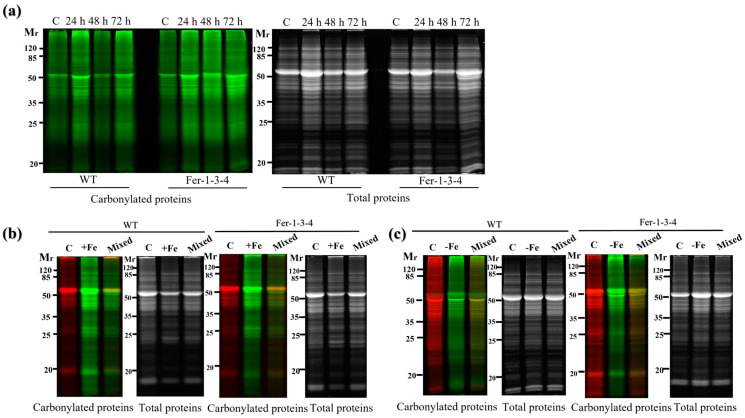

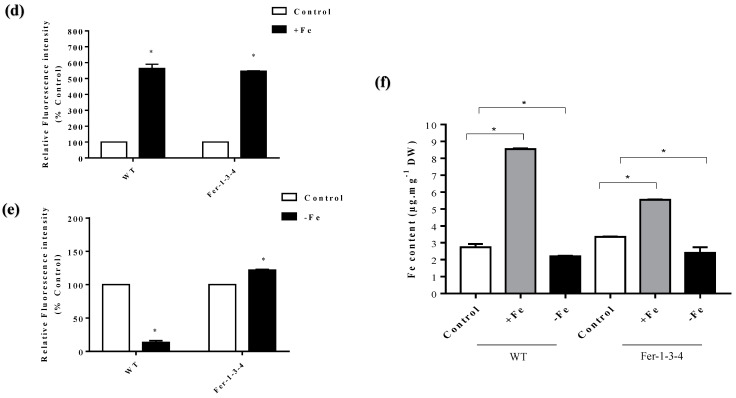
Protein carbonylation of the wild type and *Fer-1-3-4* mutant under excess iron and iron-deficient conditions. The proteins were extracted from the wild type (WT) and *Fer-1-3-4* mutant, which were grown on half MS-agar plates. (**a**) Ten days-old seedlings were transferred into a liquid MS medium supplemented with 500 µM ferric-EDTA (+Fe) for 24, 48 and 72 h. The control treatment designated by the letter C was carried out in the liquid medium without iron supplementation. The carbonylated proteins were labeled with the fluorescent hydrazide probes Cy5.5-Hz (green fluorescence), and the staining of the total proteins was carried out with AzureRed. (**b**) Exposure to excess-iron conditions. The WT and ferritin triple mutant (*Fer-1-3-4*) plants were grown on solid half-MS agar plates supplemented with 100 mM Fe-EDTA for 10 days. The letter (c) indicates the control sample from the plants grown on only solid half-MS agar without iron supplementation; the carbonylated proteins were labeled with Cy5.5-Hz. (+Fe), excess-iron treatment; the proteins were labeled with Cy7.5-Hz. (Mixed), a mixture of 5 µg of control sample labeled with Cy5.5-Hz and 5 µg of iron-treatment sample labeled with Cy7.5 hydrazide. (**c**) Iron deficiency treatment. The WT and ferritin triple mutant (*Fer-1-3-4*) plants were grown on a solid half-MS agar plate, as described in B. For iron deficiency treatment, the medium was supplemented with 300 mM of ferrozine. Each lane contains 10 µg proteins, and the staining of the total proteins was carried out with AzureRed in all the experiments. The letter (c) indicates the control sample from the plants grown on only solid half-MS agar without iron treatment; the carbonylated proteins were labeled with Cy5.5-Hz. (−Fe), iron deficiency treatment; the proteins were labeled with Cy7.5-Hz. (Mixed), a mixture of 5 µg of control sample labeled with Cy5.5-Hz and 5 µg of iron-treatment sample labeled with Cy7.5 hydrazide. Mr designates the protein molecular weight marker. (**d,e**) The abundance of carbonylated proteins based on their relative fluorescence intensity (% of control). The plotted values were obtained as described in Figure 4b,c, respectively. The experiments were performed in biological triplicate; the data points show the mean values ± standard error (n = 3), and an asterisk (*) indicates a significant difference between the control and iron stress treatment in the two genotypes (Student’s *t*-test; *p* < 0.05). (**f**) Iron content in the two genotypes under excess iron and iron deficiency treatments. The data represent the mean ± SE (n = 3) of three independent experiments. An asterisk (*) indicates a significant difference between the two genotypes (one-way ANOVA, followed by Tukey test; *p* < 0.05). DW denotes Dry weight. See Figures S5–S7 in the supplementary file for replicates of Figure 4a–c, respectively.

To examine the influence of the iron levels in the growth medium on the carbonylation of cellular proteins, the profile of the carbonylated proteins was analyzed in seedlings that were grown in a standard MS growth medium (iron-sufficient conditions) and then transferred to the same medium supplemented with 500 µM of Fe-EDTA (iron-excess condition) for 72 h. We found that the profile of the carbonylated proteins was similar between the WT and *Fer-1-3-4* mutant seedlings for that short period of exposure to excess iron in the growth medium (Figure 4a, Figure S5). However, when the seeds were sown and grown for 10 days on a solid MS medium containing excess iron, the carbonylated proteins accumulated at an important level in both WT and *Fer-1-3-4* mutant seedlings compared to the untreated seedlings (Figure 4b,d: compare the lane termed “Mixed” with the two lanes on its left, Figure S6). This indicated that the long-term exposure of Arabidopsis seedlings to excess iron promoted the carbonylation of proteins in vivo. Similarly, we found that the profile of the carbonylated proteins was altered in the WT and the *Fer-1-3-4* mutant seedlings exposed to iron deficiency for 10 days (Figure 4c,e: compare the lane termed “Mixed” with the two lanes on its left, Figure S7). Under both iron-excess and iron-deficient conditions, the profile of the carbonylated proteins differed between the WT and the *Fer-1-3-4* seedlings (Figure 4d,e, respectively). As described above, we measured the iron content of the samples to assess the link between the intracellular iron content and the level of carbonylated proteins. The iron content was slightly less in the WT than in the mutant under control conditions, but it significantly increased in both the WT and *Fer-1-3-4* mutant when exposed to excess iron, although to a lesser extent in the mutant than in the WT (Figure 4f). In contrast, we observed that the iron contents between the WT and mutant seedlings exposed to an iron deficiency treatment were significantly lower than the control, and there was no difference between the genotypes (Figure 4f). Hence, under the excess iron growth conditions, the level of carbonylated proteins increased in parallel with the increased iron content of the seedlings, whereas under iron deficiency, a few proteins became differentially carbonylated in the seedlings, even though the iron content did not change. 

**Figure 5:**
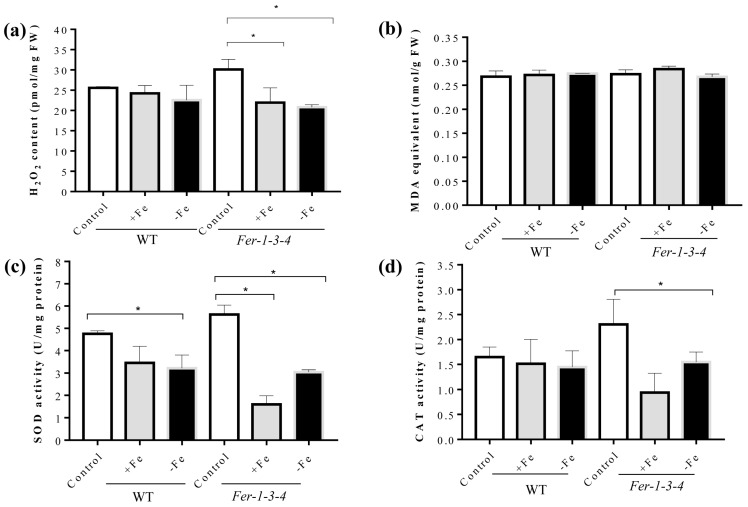
Levels of H_2_O_2_, MDA, and the antioxidant enzyme activity in the seedlings kept under excess-iron and iron-deficient conditions. The growth of the seedlings and the treatments were performed as described in Figure 4. (**a**) Hydrogen peroxide (H_2_O_2_) accumulation. (**b**) Lipid peroxidation level measured in terms of MDA using the TBAR method. (**c,d**) The measurements of SOD and CAT activity using spectrophotometry methods, respectively. Superoxide dismutase (SOD) and catalase (CAT) activity were measured on the protein extracts taken from the *Arabidopsis* wild type (WT) and *Fer-1-3-4* mutant seedlings exposed to excess iron (+Fe) or iron-deficient (−Fe) conditions for 10 days, as described above. The experiments were performed in biological triplicate, and the data points show the mean values ± standard error (n = 3). Asterisks (*) indicate significantly different values at *p* < 0.005, one-way ANOVA, and Tukey’s post hoc test. FW denotes fresh weight. See Figure S8 in the supplementary file for supporting evidence for Figure 5a.

As protein carbonylation also requires ROS in addition to iron, we examined the effects of the iron treatments on both the accumulation of hydrogen peroxide (H_2_O_2_), superoxide radicals (O_2_^−^), malondialdehyde (MDA), and on the level of superoxide dismutase and catalase activities. The H_2_O_2_ content did not significantly change in the WT, but there was a small decrease in the *Fer-1-3-4* ferritin mutant (*Fer-1-3-4*) under the excess-iron and iron-deficient conditions (Figure 5a). These observations were confirmed after staining the seedlings using 3,3′-Diaminobenzidine (DAB) and nitro blue tetrazolium chloride (NBT) to detect H_2_O_2_ and O_2_^−^, respectively (Figure S8). Consistent with the level of these ROS, there was no significant difference in the level of MDA in the WT and the mutant seedlings (Figure 5b). Moreover, SOD activity was lower under iron-excess and iron-deficient conditions than under the control condition in the WT and *Fer-1-3-4* ferritin mutant. CAT activity did not change in the WT but decreased in the *Fer-1-3-4* ferritin mutant under both iron treatments, which was in agreement with the measured H_2_O_2_ level (Figure 5c,d). These observations indicated that a change in the iron availability in the growth medium greatly influenced the occurrence of protein carbonylation in the Arabidopsis seedlings independent of ROS accumulation and oxidative stress.

### 2.4. Identification of Carbonylated Proteins in WT in Response to Iron Deficiency by LC-MS/MS Analysis

**Figure 6:**
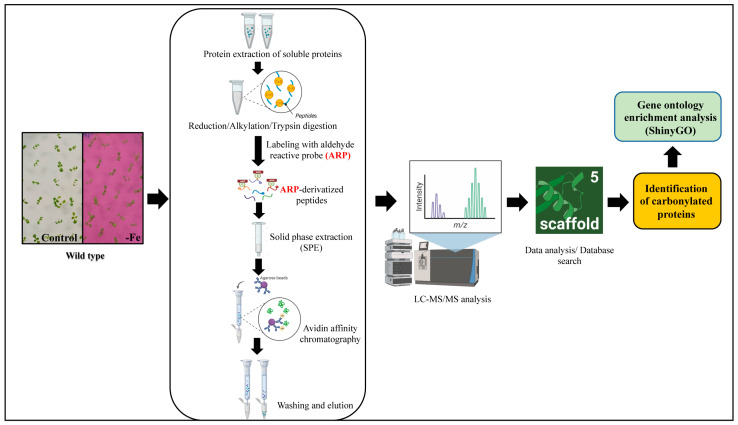
Workflow of the analysis of the carbonylated proteins via LC-MS/MS. The soluble protein extracts of the seedlings were reduced, alkylated, and digested with trypsin. To identify the carbonylated peptides, the peptides from the tryptic digestion were labeled with an aldehyde-reactive probe (ARP) for 2 h. ARP is a biotinylated hydroxylamine derivative, N′-aminooxy-methylcarbonylhydrazino-D-biotin, selectively used for the derivatization, enrichment, and mass spectrometric characterization of carbonylated proteins [55]. The hydroxylamine group of an ARP specifically forms aldoxime/ketoxime derivatives with the aldehyde and/or keto group that allows selective detection and enrichment via affinity chromatography [53,56]. Excess ARP or unbounded ARP was removed through solid phase extraction, and the ARP-derivatized peptides were enriched via biotin-avidin-based affinity chromatography, as described in the Materials and Methods section. The eluted ARP-labeled peptides were desalted and then subjected to LC-MS/MS analysis. Created with BioRender.com.

To examine whether iron promotes the carbonylation of a particular set of proteins, we sought to identify the proteins that are carbonylated in the Arabidopsis seedlings in response to iron (Fe) deficiency. We extracted the proteins and performed a peptide-level enrichment of the carbonylated proteins prior to LC-MS/MS analysis, as described previously (Figure 6) [53,54]. Additional details about the procedures that were used to identify the peptides and proteins are provided in the methods section of this article. We also looked for sites of modifications by searching for adducts with lipid-derived electrophiles malondialdehyde (MDA), 4-hydroxy-2-nonenal (HNE), acrolein, 4-oxo-nonenal (ONE), 4-hydroxy-2-hexenal (HHE), 4-oxo-2-hexenal (OHE), crotonaldehyde, cinnamaldehyde and pentanal based on the expected mass shifts when Lys, Arg, Thr, and Pro are carbonylated.

**Figure 7:**
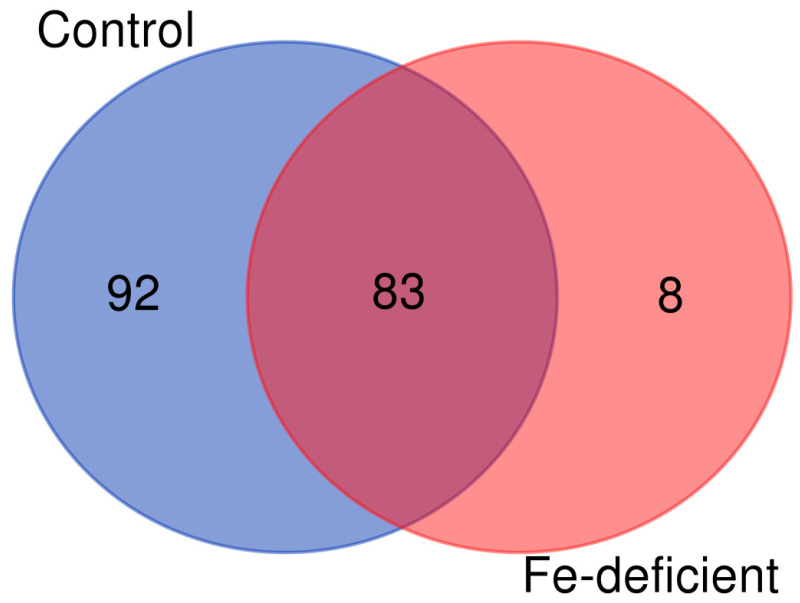
A Venn diagram of the carbonylated proteins identified in the samples derived from the control plants and the plants subjected to iron deficiency. The diagram was generated from only the carbonylated proteins identified in all three biological replicates of each group.

This approach was shown to facilitate the detection of the modification sites compared to the enrichment of the carbonylated proteins at the protein level [12,53]. A total of 183 carbonylated proteins were identified through the use of LC-MS/MS after manual data curation. Of these, 175 and 91 carbonylated proteins were found in three sample replicates of the control and iron deficiency treatment, respectively (Figure 7). 

Interestingly, eight carbonylated proteins were exclusively identified in response to iron deficiency, whereas ninety-two carbonylated proteins were only identified in the control samples (Figure 7). A few selected carbonylated proteins uniquely identified in the control or iron-deficient samples are listed in Table 1 and Table 2, respectively. The eight proteins that were found to be carbonylated specifically in the seedlings under iron-deficient conditions include the plasma membrane H+-ATPase (HA2), NADPH-protochlorophyllide oxidoreductase (PorC), receptor for activated C kinase 1A (RACK1A), nitrilase 1 (NIT1), subtilisin-like protease, 50S ribosomal protein L5, 60S ribosomal protein L15-1, and 30S ribosomal protein S2 (Table 2). The plasma membrane proton pump H^+^-ATPase (AHA2) is an important P-type proton pump that mediates the acidification of the rhizosphere in response to Fe deficiency. The acidification triggers the solubilization of Fe^3+^, which helps to free Fe^3+^ from chelated complexes prior to its reduction into Fe^2+^ and uptake into the root cells. NIT1 catalyzes the conversion of indole-3-acetonitrile to the auxin indoleacetic acid (IAA) in Arabidopsis, and Fe-deficiency increases auxin levels in the root to induce the formation of branched root hairs [57]. RACK1A is involved in multiple signal transduction pathways, and three isoforms in *A. thaliana* act redundantly to negatively regulate abscisic acid responses during seed germination and early seedling development [58,59]. Eighty-three (83) carbonylated proteins were simultaneously found in the control and iron deficiency treated plant samples (Table 3). 

In addition to identifying proteins carbonylated via direct carbonylation, we looked for modifications by searching for adducts with lipid-derived electrophiles malondialdehyde (MDA), 4-hydroxy-2-nonenal (HNE), acrolein, 4-oxo-nonenal (ONE), 4-hydroxy-2-hexenal (HHE), 4-oxo-2-hexenal (OHE), crotonaldehyde, cinnamaldehyde, and pentanal. We identified five sites modified by OHE, three sites modified by acrolein, four sites modified by ONE, two sites modified by MDA, and one site for the other carbonyl aldehydes (Table S2). A full list of all of the identified proteins in this study is available in the Supplementary Materials.

### 2.5. Protein Carbonylation Targeted Different Processes under Control and Iron-deficient Conditions

**Figure 8:**
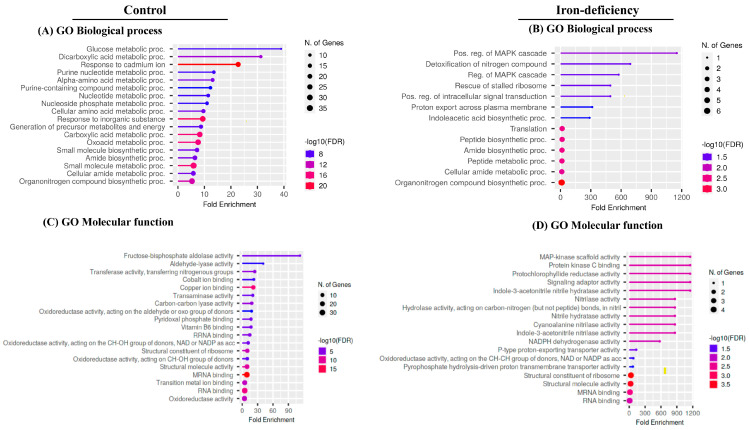
Gene ontology enrichment analysis. The bubble chart of the top 20 Gene Ontology (GO)-terms represented by the carbonylated proteins in the control plant samples and in the samples of the plants subjected to iron deficiency was generated using the ShinyGO web-based bioinformatics resource. The analysis was performed only on the carbonylated proteins identified in all three biological replicates of each treatment. (**A**,**B**) biological processes. (**C**,**D**) molecular function. The *Y*-axis represents the GO terms while the *X*-axis indicates the value of fold enrichment.

To better understand the biological processes affected by protein carbonylation in response to iron deficiency, we performed gene ontology (GO) enrichment analysis on the two lists of carbonylated samples using ShinyGO, a web-based bioinformatics resource [60]. Distinct functional categories were overrepresented in the list of carbonylated proteins derived from the Fe-deficiency-treated samples compared to the control samples (Figure 8). The processes of glucose metabolism (GO:006006), response to cadmium ion (GO:0046686), and dicarboxylic acid metabolism (GO:0043648) were more than 20-fold enriched within the 92 carbonylated proteins uniquely found in the control samples. In contrast, the biological processes related to the positive regulation of MAPK cascade (GO:0043410), the positive regulation of intracellular signal transduction (GO:0120029), proton export across the plasma membrane (GO:0120029), indoleacetic acid biosynthesis (GO:0009684), and rescue stalled ribosome (GO:0072344) were overrepresented in the list of carbonylated proteins in response to Fe deficiency. In agreement with the overrepresented biological processes, fructose–bisphosphate–aldolase activity, aldehyde–lyase activity, transferase activity, and copper and cobalt ion binding were the molecular functions overrepresented in the list of the carbonylated proteins identified in the control samples. In contrast, the enzymes having MAPK scaffold activity, protochlorophyllide reductase activity, indole-3-acetonitrile hydrase activity, nitrilase activity, and P-type proton-exporting transporting activity were overrepresented among the carbonylated proteins in response to Fe-deficient conditions. 

**Figure 9:**
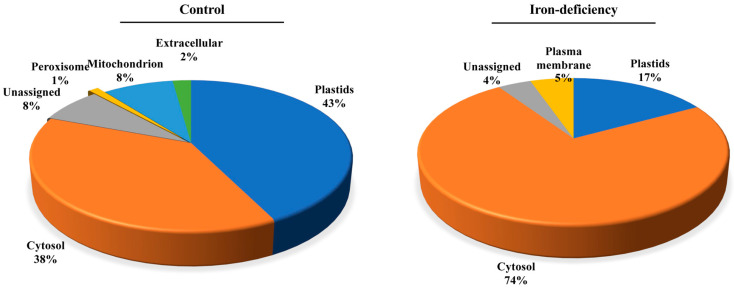
Iron prominently induced protein carbonylation in the cytoplasm. The subcellular localization of the carbonylated proteins in the control samples and the Fe-treated samples was predicted by using the Subcellular Proteomic Database, SUBA.

We predicted the subcellular localization of the identified carbonylated proteins by using the Subcellular Proteomic Database, SUBA (http://suba.plantenergy.uwa.edu.au; [61]) (Figure 9). The carbonylated proteins specific to the control samples were found to be distributed in mostly all organelles, with a significant number of them being in the chloroplasts (43%) followed by the cytosol (38%). Only 8% of them were assigned to the mitochondria (Figure 9). In comparison, the carbonylated proteins found uniquely in the Fe-deficiency-treated seedlings were mainly enriched in the cytosol (74%), and only 17% and 5% of them were assigned to the plastids and plasma membrane, respectively. It is noteworthy that no plasma membrane localization was assigned to the carbonylated proteins identified in the untreated samples, which suggests that iron deficiency may specifically direct the carbonylation of membrane proteins. We also examined the group of carbonylated proteins that were found both in the control samples and the Fe-deficiency samples. These were mostly involved in the reductive pentose–phosphate cycle (GO:0019253), carbon fixation (GO:0015977), photosynthesis, the dark reaction (GO:0019685), and the response to cadmium ions (GO:0046686) (Figure S9). The over-represented molecular functions included enzyme activator, disordered domain-specific binding, and copper ion binding. The proteins were assigned to different compartments: cytosol (27%), plastid (18%), peroxisome (5%), and mitochondria (3%).

Overall, the gene enrichment analyses indicated that iron deficiency mostly led to the carbonylation of cytosolic and plasma membrane proteins involved in regulation of intracellular signal transduction and ribosome functions, and of chloroplast proteins involved in auxin and chlorophyll metabolism. 

## 3. Discussion

In this study, we investigated how iron influences protein carbonylation in vivo. For that, we compared the carbonylated proteomes of a ferritin triple mutant (*Fer-1-3-4*) with those of the wild type under heat stress and exposure to iron deficiency and to excess iron.

### 3.1. Ferritins are More Relevant to the Steady State Occurrence of Protein Carbonylation under Normal Growth Conditions in Plant Organs

Firstly, the results of this study revealed that the major variation in the occurrence of protein carbonylation during vegetative growth is independent of stress. This agrees with the well-documented fact that under normal growth conditions, plants accumulate numerous carbonylated proteins [62,63]. In comparison to the WT, there was a difference in the pattern of carbonylated proteins observed in the ferritin mutant (*Fer-1-3-4*) during the early growth stage and toward the end of vegetative growth. This indicated that ferritin might play a role in the accumulation of carbonylated proteins during plant growth. Consistently, a difference was seen in the level of carbonylated protein in different organs of the WT and ferritin mutant (*Fer-1-3-4*). Notably, more carbonylated proteins were accumulated in the stems of *Fer-1-3-4* than in the WT. This was in accordance with reports from Ravet and colleagues that suggested the existence of different iron pools in the plant organs [47]. While the results of this study validated this thesis, they also revealed the influence of the iron pools on protein carbonylation in *A. thaliana* in vivo under normal growth conditions. Beyond this, the results strongly support the fact that iron homeostasis influences protein carbonylation under stress conditions. Previous studies have shown that ferritins are induced by excess iron or abiotic stress but repressed in the case of iron deficiency, leading to increased iron availability [34,46,47,64]. Based on that, we reasoned that the influence of iron on protein carbonylation could be brought up further when accessing the WT and the ferritin mutant under stress conditions. Not only did the heat stress increase the level of carbonylation in the plants, but the changes in the profile of carbonylated proteins were more significant in the ferritin triple mutant (*Fer-1-3-4*) than in the WT (Figure 3). Collectively, these observations revealed that intracellular iron availability influenced the occurrence of protein carbonylation, and although ferritins may not play a major role in iron storage in the plants [35,47], their disruption had an impact on protein carbonylation. Cryptic iron release by ferritins might serve to induce discrete protein carbonylation events. The question, then, was about the conditions in which iron is released by ferritins to induce carbonylation in vivo as well as the proteins of which the function is influenced by this modification.

### 3.2. The Induction of Protein Carbonylation by Intracellular Iron Is Independent of An Oxidative Stress

In this study, the result revealed that both excess iron and iron-deficient conditions impact the carbonylation of proteins, as judged through the changes in the profile of the carbonylated proteins (Figure 4). When the exposure to excess iron began from the germination, as would be the case in soils rich in iron, excess iron in the medium increased protein carbonylation in the WT and in *Fer-1-3-4*. However, the fact that *Fer-1-3-4* contained similar and sometimes fewer carbonylated proteins than WT seemed counterintuitive and might stem from a marked decrease in iron uptake by the mutant. Reduced iron uptake in *A. thaliana* exposed to excess iron has been previously reported [64]. Remarkably, the H_2_O_2_ content did not significantly change in the WT and even decreased in the ferritin mutant under both the excess-iron and iron-deficient conditions. The CAT activity was lower in the ferritin mutant, which mirrored the measured H_2_O_2_ content. Similarly, there was no significant difference in the level of MDA in the WT and ferritin mutant under both conditions. These indicated that the changes in the profiles of the carbonylated proteins observed under both the excess-iron and iron-deficient conditions would stem from the fluctuations in the iron pools rather than oxidative stress. Indeed, excess iron supply induces ferritin expression, whereas iron deficiency inhibits ferritin expression [47,65,66]. Small variations in intracellular availability likely serve to induce specific events of protein carbonylation independent of oxidative stress.

Furthermore, more carbonylated proteins were identified in the control samples than in the iron deficit-treated samples in this study. This is reminiscent of our previous study, where fewer numbers of proteins were found to be carbonylated in ABA-treated Arabidopsis seedlings than in the untreated seedlings [12]. Most of the carbonylated proteins identified in the control samples were antioxidant enzymes involved in cellular response to stress (catalase 3, monodehydroascorbate reductase 1, and thioredoxin protein), carbohydrate metabolism and energy production (fructose–bisphosphate aldolase 6, malate dehydrogenase, and phosphoglycerate kinase 3), protein folding (Heat shock 70 kDa protein and chaperonin 60), ion channel transport (V-type proton ATPase subunit B1 and C), amino acid metabolism (aspartate aminotransferase and glutamate dehydrogenase 1) and plant defence (Metacaspase-4). These observations suggest that i) numerous enzymes and stress-responsive proteins are carbonylated in the plants under control conditions in the absence of stress, which may contribute to their homeostasis, and ii) the carbonylated proteome is responsive to external cues, such as iron availability. 

### 3.3. Protein Carbonylation Appears as a Candidate Mechanism for the Control of Iron Deficiency Response in A. thaliana

The biological significance of the carbonylation of proteins under iron deficiency remains to be investigated. The findings of this study, which revealed that AHA2 and NIT1 were carbonylated under iron deficiency, appeared counterintuitive. However, evidence that heavy metal stress decreases the activity of H^+^-ATPase was reported [67], and the activity of the proton pump might be modulated at the protein level through reversible phosphorylation [68,69]. There is limited evidence concerning AHA proteins undergoing another form of PTM other than phosphorylation, but we speculate that the carbonylation of AHA2 and of NIT1 under iron deficiency may serve to modulate their activity and abundance. A quantitative proteomics approach would have helped elucidate this aspect. As for RACK1 and ribosomal proteins (Table 2), it is worth noting that the ribosomal protein L15-1 was reported to be engaged in ribosome binding to mRNA during translation [70]. The binding of the yeast RACK1 to ribosomes represents a crucial way of regulating translation and was shown to be essential for the full translation of capped mRNAs and the efficient recruitment of eukaryotic initiation factor 4E (eIF4E) [71]. Likewise, RACK1 physically interacts with Arabidopsis Eukaryotic Initiation Factor6 (eIF6), whose mammalian homolog is a key regulator of 80S ribosome assembly [72]. More than 80% of the genes co-expressed with RACK1 encode ribosome proteins, implying that RACK1 may be required for the normal production of 60S and 80S ribosomes [72]. In another study of the Arabidopsis transcriptome response to iron deficiency, a gene co-expression analysis revealed a subnetwork containing RACK1A and RACK1B and the four major regulators of Fe homeostasis, including BTS, PYE, bHLH39, and bHLH101 genes as well as several central players in Fe homeostasis, such as OPT3, FRO3, NAS4, IRT1, and MTPA2. These findings underlined the interplay between protein translation and iron homeostasis, and our current results suggest that RACK1 proteins may constitute the node between the two processes. Indeed, yeast cells were shown to activate global repression of protein synthesis after exposure to iron starvation [73]. Whether the same mechanism takes place in Arabidopsis is unknown, but we may speculate that the protein carbonylation of RACK1 and ribosomal proteins might serve to repress translation in plants under iron deficiency. 

A limitation of our study is that the results reported here do not answer the key question of the benefits or importance of protein carbonylation in terms of plant response to iron deficiency. Does the carbonylation of certain proteins promote iron uptake or repress it? Our results indicated that certain proteins involved in the iron deficiency response became carbonylated under iron-deficient conditions. However, as we did not use a quantitative proteomics approach, the proportion of the carbonylated versus non-carbonylated version of these proteins is unknown. Since carbonylation a priori inactivates a protein and leads to its degradation (as reviewed in [2]), the carbonylation of a negative regulator of iron uptake under iron deficiency would promote iron uptake. In the literature, the study of Fe-deficiency response genes uncovered BTS as a negative regulator of Fe uptake [74]. BTS is expressed throughout the plant [75,76]. Knockdown mutants of BTS accumulate significant Fe following increased Fe uptake [74]. BTS contains three hemerythrin domains that bind Fe and oxygen and lead to its degradation [75,76]. The deletion of two of these domains (1 and 2) reduces the number of Fe bound by BTS and concurrently renders BTS stable [75,76,77]. In vitro translation experiments using a wheat cell lysate showed that BTS is not targeted by the 26S proteasome since the compound of MG132, a 26S proteasome inhibitor, does not influence its stability. Moreover, in this same system, the addition of Fe leads to the degradation of BTS, while Fe chelators promote its accumulation [75,76]. These showed that the instability of BTS is triggered by the presence of Fe; we speculate that BTS could be carbonylated following iron binding. In our study, we did not identify BTS, but the detection of the full-length BTS protein is known to be very challenging [76]. The precise mode of the regulation of BTS protein level in the plant and the mechanism by which its binding to Fe leads to its degradation in the cell remain to be investigated. In our opinion, testing the hypothesis that Fe regulates its own uptake via the carbonylation of a regulatory factor such as BTS would further broaden our comprehension of the plant’s response to iron deficiency. 

## 4. Materials and Methods

### 4.1. Plant Materials and Growth Conditions

The seeds of *Arabidopsis thaliana* were ordered from the stock centre of *A. thaliana* (ABRC), and the wild type of *A. thaliana* L. (Col-0) plants were used for all experiments. The ferritin triple mutant (*Fer-1-3-4*) line was obtained as a gift from Dr Christian Dubos (Biochimie et Physiologie Moleculaire des Plantes, Montpellier, France) and was confirmed through the use of PCR (Polymerase Chain Reaction) screening using T-DNA-specific primers and gene-specific primers, as previously described [47]. The primer sets are listed in Supplementary Table S1. The seeds were surface-sterilised using 70% (*v*/*v*) ethanol for 2 min followed by 5% (*v*/*v*) NaOCl (bleach) containing 0.02% SDS (Sigma-Aldrich, St. Louis, MS, USA) for 3 min and then rinsed four times with sterile water. The seeds were placed onto half-strength Murashige and Skoog (MS) medium (Phytotechnology Laboratories, Shawnee Mission, KS, USA) supplemented with 0.7% agar and 1% sucrose (pH 5.8) then stratified for 2 days at 4 °C in the dark before the plates were transferred to a growth chamber and grown at 22 °C in a photoperiod of 14 h darkness followed by 10 h of light [78].

### 4.2. Profiling of Total Carbonylation Levels at Different Growth Phases and in Different Organs of Arabidopsis Plants

To profile the carbonylated proteins within the wild type (WT) and *Fer-1-3-4* mutant, the plants were grown for 6 weeks after stratification, and the leaves were collected at 7, 14, 28, and 42 days of plant growth, corresponding to the principal growth stage of 1.0 (early vegetative phase), stage 1.04 (leaf development), stage 3.70 (rosette growth), and stage 6.50 (flower production), respectively, as described by [50]. The leaf samples were harvested under liquid nitrogen prior to storage at −80 °C before protein extraction. Three independent experiments were conducted. To examine the carbonylated protein levels in the different organs of the plant, leaves, stems, and flowers were harvested from six-week-old plants grown on soil at 22 °C with 14 h dark/10 h light period in a controlled growth chamber. The samples were harvested in liquid nitrogen prior storage at −80 °C before protein extraction and three independent experiments were conducted with WT and *Fer-1-3-4*.

### 4.3. Heat Stress Treatment 

The seedlings were initially grown on half-strength MS plates for 10 days before they were subjected to the heat treatment that consisted of moving the plates at 38 °C for 1 h, 3 h, and 6 h in an incubator in the dark. Then, recovery was performed in a growth chamber at 22 °C for 2 h under a light, as previously described [78]. The control plates were kept at 22 °C under the same conditions. Three different plates of seedlings that were grown under light for 10 days were used in each experiment.

### 4.4. Iron Treatments 

For long-term exposure to excess iron, the seeds were grown on solid half-strength MS medium supplemented with 500 µM Fe (III)-EDTA (Alfa Aesar IL, USA) at 22 °C with 14 h dark/10 h light period for 10 days [64,79]. For short-term excess iron treatment, the seedlings were pre-cultivated on half-strength MS medium for 10 days and then transferred into fresh (^1^/_2_ MS) liquid medium supplemented with 500 µM Fe (III)-EDTA for 24 h, 48 h, and 72 h. 

For the iron deficiency treatment, the seeds were grown on a half-strength MS plate containing 300 µM 3-(2-pyridyl)-5-6-diphenyl-1,2,4-triazine sulfonate (ferrozine) (Alfa Aesar, Ottawa, ON, Canada). The control experiment was undertaken by growing the seeds on solid half-strength MS medium plates for 10 days [75]. The seedlings were harvested under liquid nitrogen prior to storage at −80 °C before protein extraction. Three independent experiments were conducted.

### 4.5. H_2_O_2_ Content Estimation

The hydrogen peroxide (H_2_O_2_) content was measured using the 2,2′-azino-bis (3-ethylbenzthiazoline-6-sulfonic acid) diammonium salt (ABTS; Sigma Aldrich, St. Louis, MO, USA) method, as previously described [80], with slight modifications. Briefly, ten-days-old wild-type and *Fer-1-3-4* seedlings grown under excess iron and iron-deficient conditions were harvested, and the H_2_O_2_ from the plant extracts was extracted, as described in [81]. H_2_O_2_ production was revealed through the use of ABTS, which reacts with H_2_O_2_ in the presence of horseradish peroxidase (HRP) to generate a soluble end product (radical monocation ABTS**·**+). The radical monocation ABTS**·**+ was detected spectrophotometrically at 405 nm [80].

### 4.6. DAB and NBT Staining Procedures

3,3-diaminobenzidine (DAB) staining was used to estimate H_2_O_2_ accumulation in the seedlings of WT and *Fer-1-3-4*, as previously described [82,83]. Briefly, the seedlings were incubated in freshly prepared DAB staining solution (1 mg/mL DAB and 0.1% Tween 20 in 10 mM Na_2_HPO_4_ for 8 h and further rinsed with 70% ethanol several times to remove chlorophyll.

Nitrotetrazolium blue chloride (NBT) staining was used to detect superoxide radical content (O_2_^−^), as previously described [84,85]. The seedlings were incubated in NBT staining solution (0.2% NBT in 50 mM sodium phosphate buffer pH 7.5) for 5 h and further rinsed with 70% ethanol several times to remove chlorophyll. The images of the seedlings were captured using a digital camera.

### 4.7. Lipid Peroxidation Assay

For the measurement of the lipid peroxidation products, the formation of malonaldehyde (MDA) was estimated in the form of TBA-reactive substance (TBARS) and the difference of absorbance of 532 nm and 600 nm using the extinction coefficient of 155 mM^−1^ cm^−1^ [86].

### 4.8. Antioxidant Enzyme Activity of Catalase (CAT) and Superoxide Dismutase (SOD)

Ten-days-old *Arabidopsis* seedlings grown on half-strength MS medium plates were harvested and homogenized in cold 100 mM phosphate buffer pH 7 containing 1 mM EDTA and 1% polyvinylpyrrolidone and then centrifuged for 10 min at 10,000× *g* at 4 °C to eliminate plant debris. One hundred milligrams of plant fresh weight (FW) were used for one milliliter of phosphate buffer. The supernatants were collected and used for antioxidant enzyme activity within 4 h. The protein content in the supernatant was estimated using the Bradford assay.

Catalase (CAT) is a common and highly active enzyme that catalyzes the decomposition of H_2_O_2_ to nontoxic water and O_2_ [87]. CAT activity was measured in 50 mM phosphate buffer pH 7.0 containing 1 mM EDTA, 3% H_2_O_2_, and crude enzyme solution (the supernatant), as previously described [88]. The decrease in absorbance at 240 nm was monitored for 1 min and the CAT activity was presented as µmol H_2_O_2_ decomposed min^−1^ (unit) mg^−1^ protein.

Superoxide dismutase (SOD) is one of the important antioxidant enzymes that catalyzes the dismutation of the superoxide anion into hydrogen peroxide and molecular oxygen. SOD activity was analyzed through measuring the ability to inhibit the photochemical reduction of NBT at 560 nm. The reaction mixture was composed of 100 mM phosphate buffer with pH 7.8, 1 mM EDTA, 130 mM L-methionine, 750 µM NBT and 20 µM riboflavin, and 50 µL of crude enzyme extract (supernatant). The samples were illuminated for 15 min, the absorbance was measured at 560 nm, and the amount of SOD corresponding to 50% of the reaction was defined as one unit of the enzyme [88].

### 4.9. Protein Extraction

The plant protein extraction buffer comprised 50 mM Tris-HCl (pH 8.0) buffer, 0.1% triton X-100, 10 mM EDTA, 50 mM DTT, and 1:100 protease inhibitor. The plant seedlings or leaves were ground into a powdered form using a precooled mortar and pestle under liquid nitrogen prior to storage at −70 °C before protein extraction. The powdered samples were resuspended in the extraction buffer and centrifuged at 20,000× *g* for 10 min at 4 °C. Afterward, the supernatant was transferred to clean tubes for the subsequent experiments. The total crude protein concentrations (µg/µL) in the extracts were estimated using the Bradford method [89]. Briefly, 5 µL of protein sample was mixed into 1 mL of Bradford reagent (Bio-Rad Protein, Assay) and incubated for 5 min at RT. Absorbance at 595 nm was measured with the aid of a spectrophotometer (UV-VIS 1280 Spectrophotometer, Shimadzu, Japan).

### 4.10. Derivatization of Total Protein with Fluorescent Hydrazide Dye (Cy5.5 and Cy7.5)

This derivatization method was modified from the protocol previously described by Tamarit et al. [90]. Prior to the derivatization assay, 10 mM stock solution of the dyes was prepared in DMSO from 1 mg fluorescent hydrazide dye powder and then tenfold diluted in 0.1 M sodium acetate with pH 5, 1 mM EDTA and 1% SDS as the corresponding hydrazine working solution. One hundred microliters of Cy5.5-hydrazide working solution was added to 100 µL of total protein sample containing 100 µg of total crude protein (control samples). In parallel, 100 µL of Cy7.5-hydrazide solution was also added to 100 µL of the protein sample containing 100 µg of protein (experimental test sample). Each of the mixtures was incubated at 22 °C and continuously shaken at 500 rpm for 1 h of incubation. Then, 55 µL of 50 mM Phosphate saline buffer (PBS (phosphate-buffered saline)) with pH 7.4 and 15 µL of 0.2 M NaCNBH_3_ (Thermofisher) were added to stop the reaction, and finally, the mixtures were incubated at room temperature for 15 min. The reaction mixture was further precipitated with 270 µL of 20% trichloroacetic acid (TCA) and kept for 30 min on ice or overnight at 4 °C. The mixture was centrifuged at 10,000× *g* for 5 min, and the supernatants were discarded. Moreover, the pellets were further briefly sonicated for 3 sec (Eppendorf) and washed thrice with 1 mL of 1:1 (*v*/*v*) ethanol: ethyl acetate and centrifuged at 10,000× *g* for 10 min at 4 °C. After a final washing, the pellets were air-dried and resuspended in a protein extraction buffer with continuous pipetting and spin-down. Thereafter, the supernatant was collected and stored at −80 °C.

### 4.11. Gel Electrophoresis of Fluorescent Labeled Proteins (1D-PAGE)

For 1D-gel electrophoresis, 10 µg labeled protein were mixed with 4X Laemmli sample buffer (50 mM Tris-HCl pH 6.8, 2% SDS, 10% (*v*/*v*) glycerol, 100 mM DTT, and 0.1% bromophenol blue) and analyzed using SDS-PAGE (4% stacking gel and 12.5% resolving gel) after heating the samples at 95 °C for ~5 min. The samples were then initially run at 10 mA for 1 h, and then the current was increased to 20 mA, and the gels were run on the same current until the tracking dye crossed the gels using a vertical Bio-Rad Mini-Protean Tetra Cell.

### 4.12. In-gel Fluorescence-based Detection of the Total Proteins and the Carbonylated Proteins 

The SDS-PAGE gels were stained with AzureRed (Azure Biosystems, Dublin, CA, USA) to visualize the total protein by following the protocol of the manufacturers. The gel was first fixed in fixing solution (1.01 g of AzureRed powder A in 85 mL of ethanol and 15 mL) for 1 h with gentle rocking and further stained with Azure staining solution 1:200 (AzureRed dye: staining buffer (2.34 g of AzureRed powder B in 100 mL of sterile water) for 1 h with gentle rocking. Then, the gel was briefly washed with sterile water and washing solution (85 mL of sterile water and 15 mL of 100% ethanol) for 30 min. The gel was then removed and incubated in a fixing solution for 30 min with gentle rocking before visualization. The carbonylated proteins on the gel were visualized through the use of fluorescence imaging using the Azure Sapphire^TM^ Biomolecular imager (Azure Biosystems, CA) with the following specific settings: 100 µm pixel size for resolution scan, 658 nm (red color) for Cy5.5, and 784 nm (green color) near-infra-red for Cy7.5). The total protein was detected at 520 nm. The gel images were visualized using Azure Sapphire^TM^ Biomolecular imager (Azure Biosystems, CA) and the fluorescent signal intensity was analyzed using AzureSpot (Azure Biosystems, CA). For quantification, the total fluorescent signal intensity was obtained from each lane after background subtraction across the gel was performed, and the total protein signal intensity was also measured from the entire lane. Then, the fluorescence signal intensity (total carbonyl content) was normalized to the total protein signal intensity.

### 4.13. Determination of Iron (Fe) Content

The total iron content in the plant tissues (leaves, stems, flowers, and seedlings) was determined using a procedure previously described by [51]. Plant tissues, including the leaves, stems, flowers, and seedlings (10-day-old seedlings), were collected and rinsed with de-ionized water and further oven-dried at 65 °C for 2 days. Then, the dried tissues were digested with 65% (*v*/*v*) HNO_3_ at 95 °C for 6 h, followed by 30% (*v*/*v*) H_2_O_2_ at 56 °C for 2 h. The iron content in the different samples was analyzed in a mixture of digested samples and assay solution containing 1 mM bathophenanthroline disulfonate (BPDS), 0.6 M sodium acetate, and 0.48 M hydroxylamine hydrochloride [52]. The absorbance of the resulting Fe^2+^-BPDS3 complex was measured at 535 nm using a microplate reader (Synergy, USA). The standard curve was prepared using FeCl_3_ as a standard solution, and the Fe content in different samples was calculated by plotting the absorbance values against the standard curve.

### 4.14. Identification of Carbonylated Proteins and Carbonylated Sites

This method was previously described by [53]. The total crude proteins were extracted from the control and iron-deficient samples of the wild-type seedlings. For trypsin digestion, the samples were denatured with 1% (*w*/*v*) deoxycholate sodium salt (Fischer Scientific, Belgium) and further reduced with 5 mM Tris-(2-carboxyethyl)-phosphine (TCI, Miami, FL, USA) at 60 °C for 30 min with constant shaking at 500 rpm. The samples were further alkylated with 10 mM iodoacetamide (Alfa Aesar, CA) at 37 °C for 30 min in the dark. Then, the digestion was performed using 2 µg of Trypsin Gold (Promega) for 16 h at 37 °C with constant shaking at 500 rpm. The digestion was stopped with 1% formic acid, and the resulting peptides were dried under a speed vacuum. To identify the carbonylated peptides, we followed the procedure previously used in our laboratory [91,92]. Briefly, equal volumes of the peptides from the tryptic digestion were labeled with 10 mM *O*-(biotinylcarbozoylmethyl) hydroxylamine (ARP (aldehyde-reactive probe); Dojindo Laboratories) at 27 °C for 2 h. ARP is a biotinylated hydroxylamine derivative, N′-aminooxy-methylcarbonylhydrazino-D-biotin, selectively used for the derivatization, enrichment, and mass spectrometric characterization of carbonylated proteins [55]. The hydroxylamine group of an ARP specifically forms aldoxime/ketoxime derivatives, with the aldehyde and/or keto group and the intrinsic biotin tag facilitating the good stability of oximes that allows selective detection and enrichment through affinity chromatography and is generally used in LC-MS/MS-based studies for the effective derivatization of carbonylated peptides or proteins [53,56]. Unbound ARP molecules or excess ARP were then removed from the medium through the use of the solid-phase separation method with C18 Agilent columns according to the manufacturer’s recommendations (Agilent Technologies). The labeled peptides were eluted with 70% (*v*/*v*) acetonitrile and 0.5% (*v*/*v*) formic acid and dried under a speed vacuum. To achieve peptide-level enrichment, ARP-labeled peptides were purified via affinity chromatography using a monomeric avidin agarose slurry (Thermo Scientific) according to the manufacturer’s instructions and eluted with 70% (*v*/*v*) acetonitrile and 0.5% (*v*/*v*) formic acid. The peptide solution was desalted and dried under a speed vacuum before LC-MS/MS. 

Mass spectrometry (MS) analyses were performed using the Proteomics Platform of the CHU de Québec Research Center (Quebec, QC, Canada). The samples were analyzed via nano LC/MSMS using a Dionex UltiMate 3000 nanoRSLC chromatography system (Thermo Fisher Scientific) connected to an Orbitrap Fusion mass spectrometer (Thermo Fisher Scientific, San Jose, CA, USA). The peptides were trapped at 20 μL/min in a loading solvent (2% acetonitrile, 0.05% TFA) on a 5 mm × 300 μm C18 pepmap cartridge pre-column (Thermo Fisher Scientific/Dionex Softron GmbH, Germering, Germany) for 5 min. Then, the pre-column was switched online with a Pepmap Acclaim column (ThermoFisher) 50 cm × 75 µm internal diameter separation column and the peptides were eluted with a linear gradient from 5 to 40% solvent B (A: 0.1% formic acid, B: 80% acetonitrile, and 0.1% formic acid) for 90 min at 300 nL/min for a total run time of 120 min. The mass spectra were acquired using a data-dependent acquisition mode using Thermo XCalibur software version 4.3.73.11. Full-scan mass spectra (350 to 1800 *m*/*z*) were acquired in the orbitrap using an AGC target of 4e5, a maximum injection time of 50 ms, and a resolution of 120,000. Internal calibration using lock mass on the *m*/*z* 445.12003 siloxane ion was used. Each MS scan was followed with an MS/MS fragmentation of the most intense ions for a total cycle time of 3 s (top speed mode). The selected ions were isolated using the quadrupole analyzer in a window of 1.6 *m*/*z* and fragmented through higher-energy collision-induced dissociation (HCD), with 35% collision energy. The resulting fragments were detected through the use of a linear ion trap at a rapid scan rate with an AGC target of 1e4 and a maximum injection time of 50 ms. Dynamic exclusion of previously fragmented peptides was set for a period of 20 sec and a tolerance of 10 ppm.

### 4.15. Database Searching and Criteria Used for Protein Identification and Data Analysis

Mascot generic file format (MGF) peak list files were created using Proteome Discoverer 2.3 software (Thermo). The MGF sample files were then analyzed using Mascot (Matrix Science, London, UK; version 2.5.1). Mascot was set up to search a contaminant database, and the Uniprot Reference *Arabidopsis thaliana* database (September 2020), containing 39,449 entries, was set up to evaluate the assumed samples digested with the enzyme trypsin. Mascot was searched with a fragment ion mass tolerance of 0.60 Da and a parent ion tolerance of 10.0 PPM. Carbamidomethyl of cysteine was specified in Mascot as a fixed modification. The deamidation of asparagine and glutamine and the oxidation of methionine were specified in Mascot as variable modifications. Two missed cleavages were allowed. 

Scaffold (version Scaffold_5.0.1, Proteome Software Inc., Portland, OR, USA) was used to validate the MS/MS-based peptide and protein identifications. A false discovery rate of 1% was used for the peptides and proteins. The proteins that contained similar peptides and could not be differentiated based on MS/MS analysis alone were grouped to satisfy the principles of parsimony. The analysis results were filtered according to the following set of criteria: a significant threshold of *p* > 0.05 (with a 95% confidence for protein probability score), the protein required to be present in at least one replicate and the minimum number of unique peptides was set to one, and the maximum false discovery rate (FDR) was set to 1%. 

### 4.16. Gene Ontology Enrichment Analysis (GO Analysis) 

The carbonylated proteins identified in the proteome of the control and iron-deficient samples of the WT were analyzed for different gene ontology (GO) terms (biological function, cellular distribution, and molecular function) using the ShinyGO version 0.76 database (www.bioinformatics.sdstate.edu/go/). We filtered the results by using the expressed genome of *Arabidopsis thaliana* as a reference set to obtain the annotation of the proteins. A *p*-value of ≤0.05 (FDR) was used as the threshold to determine the significant enrichment of the GO groups.

### 4.17. Statistical Analysis

The total protein carbonylation of the wild-type and *Fer-1-3-4* plants has been compared between treatments and between genotypes using a two-way analysis of variance (ANOVA) and Tukey’s multiple comparison post-test. When multiple *t*-tests were performed, the Bonferroni–Dunn method was used to calculate the adjusted *p*-value. Differences were considered to be significant when *p* < 0.05, and the statistical analyses were performed using GraphPad Prism 7.0 (GraphPad Software Inc., San Diago, CA, USA).

## 5. Conclusions

The data presented in this study indicate that intracellular iron influences protein carbonylation in *A.thaliana* in vivo, and iron deficiency triggers the carbonylation of cytosolic and plasma membrane proteins involved in signalling, protein synthesis, auxin, and chlorophyll metabolism, and the response to iron deficiency. Furthermore, our results showed that the occurrence of protein carbonylation is influenced by iron availability and ferritins, even under normal growth conditions. Protein carbonylation may represent a node linking iron deficiency response to translation and iron homeostasis. 

## Figures and Tables

**Table 1:** List of selected carbonylated proteins specifically identified in the control samples.

SN	Protein Name	Accession Number	TAIR Gene Code	Molecular Mass (kDa)	Peptide Number	Localization	Functional Category
1	Catalase 3	B9DG18	AT1G20620	56	12	Peroxisome	ROS scavenging/stress defense response/Antioxidant enzymes
2	Monodehydroascorbate reductase 1	Q9LFA3	AT3G52880	46	7	Peroxisome	
3	Thioredoxin superfamily protein	F4JBC9	AT3G26060	24	7	Chloroplast	
4	NAD(P)H dehydrogenase (quinone) FQR1	Q9LSQ5	AT5G54500	22	2	Chloroplast	
5	Alcohol dehydrogenase class-3	Q96533	AT5G43940	41	10	Cytosol	
6	V-type proton ATPase subunit B1	P11574	AT1G76030	54	9	Plasma membrane	Ion channel transport
7	V-type proton ATPase subunit C	Q9SDS7	AT1G12840	43	4	Plasma membrane	
8	Heat shock 70 kDa protein 9	Q8GUM2	AT4G37910	73	6	Mitochondria	Protein folding
9	Chaperonin 60 subunit beta 1	P21240	AT1G55490	64	15	Chloroplast	
10	Peptidyl-prolyl cis-trans isomerase CYP18-4	Q42406	AT4G34870	18	9	Cytosol	
11	Ferredoxin--nitrite reductase	Q39161	AT2G15620	66	6	Chloroplast	Nitrate assimilation/Nitrogen metabolism
12	Glutamate synthase 1 [NADH]	Q9LV03	AT5G53460	242	11	Chloroplast	Amino acid metabolism
13	Aspartate aminotransferase	P46643	AT2G30970	48	7	Mitochondria	
14	Glutamate dehydrogenase 1	Q43314	AT5G18170	45	5	Chloroplast	
15	Cysteine synthase	P47999	AT2G43750	42	7	Chloroplast	
16	Imidazole glycerol phosphate synthase hisHF	Q9SZ30	AT4G26900	64	6	Chloroplast	
17	Phosphoglycerate kinase 3	Q9SAJ4	AT1G79550	42	10	Cytosol	Carbohydrate metabolism/Calvin cycle/
18	Glucose-6-phosphate 1-dehydrogenase	A0A1I9LLZ6	AT3G27300	67	3	Cytosol	
19	Fructose-bisphosphate aldolase 6	Q9SJQ9	AT2G36460	38	5	Cytosol	
20	Triosephosphate isomerase	Q9SKP6	AT2G21170	33	8	Chloroplast	
21	Malate dehydrogenase 1	Q9ZP06	AT1G53240	36	7	Mitochondria	TCA cycle/Energy metabolism
22	Adenylyl cyclase-associated protein	A0A1P8B7W9	AT4G34490	58	3	Chloroplast	Signalling
23	Metacaspase-4	O64517	AT1G79340	45	2	Cytosol	Plant defense
24	Biotin carboxylase	F4JYE0	AT5G35360	61	9	Chloroplast	Fatty acid metabolism
25	Formate dehydrogenase	A0A1P8B9L1	AT5G14780	41	4	Mitochondria	

**Table 2:** List of carbonylated proteins identified in the iron-deficient samples only.

SN	Protein Name	Accession Number	TAIR Gene Code	Molecular Mass (kDa)	Peptide Number	Localization	Functional Category
1	Receptor for activated C kinase 1 (RACK1)	O24456	AT1G18080	36	8	Cytosol	Signal transduction
2	60S Ribosomal protein L15-1 (RPL15)	O23515	AT4G16720	24	4	Ribosome	Translation
3	50S Ribosomal protein L5 (RPL5)	O04603	AT4G01310	28	6	Ribosome	Translation
4	Nitrilase 1 (NIT1)	P32961	AT3G44310	38	6	Cytosol	Indole acetic acid biosynthesis process
5	Subtilisin-like protease (SBT1)	Q9LVJ1	AT3G14067	82	2	Cytosol	Proteolysis
6	Plasma membrane ATPase (HA2)	F4JPJ7	AT4G30190	108	4	Plasma membrane	Proton (H^+^) transmembrane transport
7	30S Ribosomal protein S2 (RPS2)	P56797	ATCG00160	27	4	Ribosome	Translation
8	NADPH-protochlorophyllide oxidoreductase (POR)	F4I2F8	AT1G03630	44	2	Chloroplast	Chlorophyll biosynthesis

**Table 3:** List of selected carbonylated proteins identified both in the control samples and iron-deficient samples.

SN	Protein Name	Accession Number	Tair Gene Code	Molecular Mass (kDa)	Peptide Number	Functional Category
1	Ascorbate peroxidase	F4HU93	AT5G02500	28	9	Stress defense response/Antioxidant enzymes
2	Peroxidase 32	Q9LHB9	AT3G32980	39	7	
3	Monodehyroascorbate reductase 6	F41576	AT1G63940	52	14	
4	Selenium binding protein 2	Q93WNO	AT4G14040	54	6	Metal-binding proteins
5	Magnesium chelatase subunite ChII-2	Q5XF33	AT5G45930	46	3	
6	Glutamate--glyoxylate aminotransferase 1	Q9LR30	AT1G23310	53	18	Glyoxylate metabolism
7	Oxalate--CoA ligase	Q9SMT7	AT3G48990	56	7	
8	Transketolase	F4JBY2	AT3G60750	80	19	Carbohydrate/Energy metabolism
9	Pyruvate kinase	Q94KE3	AT3G52990	57	7	
10	Sedoheptulose-1,7-bisphosphatase	P46283	AT3G55800	42	10	
11	Glucose-1-phosphate aldenyltransferase large subunit 1	P55229	AT5G19220	58	10	
12	Phosphoribulokinase	P25697	AT1G32060	44	9	
13	Cytosolic isocitrate dehydrogenase [NADP]	Q9SRZ6	AT1G65930	46	17	
14	Ferrodoxin-dependent glutamate synthase 1	Q9ZNZ7	AT5G04140	177	19	Nitrogen–carbon metabolism
15	Nitrile specifier protein	Q9SDM9	AT3G16400	52	16	
16	Glutamate-1-semialdehyde-2-aminomutase 1	P42799	AT5G63570	50	7	
17	Glutamine synthetase	Q43127	AT3G55800	47	12	
18	S-adenoyl-L-methionine-dependent methyltrnsferase	AOA1P8B3H0	AT4G34050	32	4	Amino acid metabolism
19	S-adenoylmethionine synthase 4	Q9LUT2	AT5G20720	43	10	
20	Serine hydroxymethyltransferase 1	Q9SZJ5	AT4G37930	57	10	
21	Glycine dehydrogenase (decarboxylating) 1	Q94B78	AT4G33010	113	17	
22	Chaperonin 60 subunit alpha 1	P21238	AT3G09260	62	17	Protein folding
23	PYK10-binding protein 1	O04314	AT4G14880	32	10	
24	Elongation factor Tu	P17745	AT4G20360	52	11	Translation/Protein synthesis
25	Elongation factor G	Q9SI75	AT1G62750	86	9	
26	Tubulin alpha-2 chain	B9DGT7	AT1G50010	50	12	Cytoskeleton organization
27	Actin-7	P53492	AT5G09810	42	7	

## Data Availability

The datasets generated during and/or analyzed during the current study are available from the corresponding author upon reasonable request. The proteomics data underlying this article are available in the article and in its online Supplementary Materials. The data can also be found here: MassIVE MSV000090535, ProteomeXchange PXD037445.

## Supplementary Materials

The following supporting information can be downloaded at: https://www.mdpi.com/article/10.3390/ijms24119732/s1.
