# Supplementary material for: Iron Availability Influences Protein Carbonylation in Arabidopsis thaliana Plants
Source: Int J Mol Sci. 2023 Jun 4;24(11):9732. doi: 10.3390/ijms24119732 (PMC10253855; doi:10.3390/ijms24119732)
Supplement: Supplementary file 1 [file ijms-24-09732-s001.zip › Supplemental Table S1.pdf]

Supplemental Table S1. Primers used in this study.

Primers	Sequence
AtFer-1 (F)	5’CAGAGCAGTGGTCGTTGCAG’3
AtFer-1 (R)	3’AGACAGAGCCAACTCCATTGC’5
AtFer-3 (F)	5’CCGGCAATCTCTCTTTCCCTC’3
AtFer-3 (R)	3’ACACTGTGCAGGTTTAGGAGC’5
AtFer-4 (F)	5’TCATCTTCTTCTTCAGCTCTCTCG’3
AtFer-4 (R)	3’TCTCCAGTGACAGAGCAAGC’5
T-DNA line from SALK institute for *fer-1* (SALK_055487C) and *fer-4* (SALK_068629C)	prok2-LBb1 (5’-GCGTGGACCGTTGCTGCAACT-3’) for Salk lines
T-DNA line from GABI Program for *fer-3* (GABI-KAT-496A08)	GABI-KAT (PAC161) (5’ATAATAACGCTGCGGACATCTACATTTT-3’) for GABI line
