# Supplementary material for: Iron Availability Influences Protein Carbonylation in Arabidopsis thaliana Plants
Source: Int J Mol Sci. 2023 Jun 4;24(11):9732. doi: 10.3390/ijms24119732 (PMC10253855; doi:10.3390/ijms24119732)
Supplement: Supplementary file 1 [file ijms-24-09732-s001.zip › Supplementary Figure captions_IJMS.pdf]

Supplementary Figure captions – Corrected.

Supplemental Figure 1: Representative gel pictures from two additional experimental replicates of the carbonylated proteins in the leaves of the wild-type (WT) plants and of the ferritin mutant plants Fer-1-3-4 over their growth period. Proteins were extracted from leaf tissues sampled from soil-grown WT and triple ferritin mutant Fer-1-3-4 plants over 42 days (6 weeks) after seed stratification. Ten micrograms of proteins were labelled with the fluorescent hydrazide probe (Cy5.5-Hz) specific for the carbonylated proteins and then analyzed on 12.5 % SDS-PAGE. Upper gel: Cy5.5-Hz fluorescence for the carbonylated proteins; lower gel: Azure red fluorescence for the total proteins. Mr designates the protein molecular weight marker. See Figures 1a and 1b in the main text.

Supplemental Figure 2: Representative gel pictures from two additional experimental replicates of the carbonylated proteins in the different organs of the wild-type (WT) plants and of the ferritin mutant plants Fer-1-3-4. The proteins were extracted from leaves, stems, and flowers of six-week-old soil-grown WT and triple ferritin mutant Fer-1-3-4 plants. The carbonylated proteins in the WT sample and the Fer-1-3-4 sample were labeled with the fluorescent hydrazide probes Cy5.5-Hz (green fluorescence) and Cy7.5-Hz (red fluorescence, pseudocolor), respectively. The samples were then analyzed on 12.5 % SDS-PAGE. Each lane contains 10 µg proteins. W: WT sample; F: Fer-1-3-4 sample; W+F: mixture of equal amount of WT and Fer-1-3-4 sample. Upper gel: Cy5.5-Hz and Cy7.5-Hz fluorescence for the carbonylated proteins; lower gel: Azure red fluorescence for the total proteins. Mr designates the protein molecular weight marker. See Figures 2a and 2b in the main text.

Supplemental Figure 3: Representative gel pictures from two additional experimental replicates of the carbonylated proteins in the wild type (WT) under heat stress. The proteins were extracted from the wild type (WT) germinated on half MS-agar plates for 10 days. The seedlings were then exposed to high temperature (38 ℃) in the dark for 1, 3, and 6 h. Control (C) plants were kept in the growth chamber at 22 ℃. After the heat treatment, plants were returned to the growth chamber at 22 ºC for 2 h before sampling. The carbonylated proteins in the control sample and the heat-treated plant samples were labeled with the fluorescent hydrazide probes Cy5.5-Hz (red fluorescence, pseudocolor) and Cy7.5-Hz (green fluorescence), respectively. The samples were then analyzed on 12.5 % SDS-PAGE, either alone or mixed in equal amounts. Each lane contains 10 µg proteins. Gel on the left: Cy5.5-Hz and Cy7.5-Hz fluorescence for the carbonylated proteins; gel on the right: Azure red fluorescence for the total proteins. Mr designates the protein molecular weight marker. See Figure 3a in the main text.

Supplemental Figure 4: Representative gel pictures from two additional experimental replicates of the carbonylated proteins in the Fer-1-3-4 mutant under heat stress. The proteins were extracted from the Fer-1-3-4 mutant germinated on half MS-agar plates for 10 days. The seedlings were then exposed to high temperature (38 ℃) in the dark for 1, 3, and 6 h. Control (C) plants were kept in the growth chamber at 22 ℃. After the heat treatment, plants were returned

to the growth chamber at 22 ºC for 2 h before sampling. The carbonylated proteins in the control sample and the heat-treated plant samples were labeled with the fluorescent hydrazide probes Cy5.5-Hz (red fluorescence, pseudocolor) and Cy7.5-Hz (green fluorescence), respectively. The samples were then analyzed on 12.5 % SDS-PAGE, either alone or mixed in equal amounts. Each lane contains 10 µg proteins. Gel on the left: Cy5.5-Hz and Cy7.5-Hz fluorescence for the carbonylated proteins; gel on the right: Azure red fluorescence for the total proteins. Mr designates the protein molecular weight marker. See Figure 3a in the main text.

Supplemental Figure 5: Representative gel pictures from two additional experimental replicates of the carbonylated proteins in the wild type and Fer-1-3-4 mutant after a short exposure to excess-iron conditions. The proteins were extracted from the wild type (WT) and Fer-1-3-4 mutant, which were grown on half MS-agar plates. Ten days-old seedlings were transferred into a liquid MS medium supplemented with 500 µM ferric-EDTA (+Fe) for 24, 48 and 72 h. The control treatment designated by the letter C was carried out in the liquid medium without an iron supplement. The carbonylated proteins were labeled with the fluorescent hydrazide probes Cy5.5-Hz (green fluorescence) and the staining of the total proteins was carried out with Azure red. Mr designates the protein molecular weight marker. See Figure 4a in the main text.

Supplemental Figure 6: Representative gel pictures from two additional experimental replicates of the carbonylated proteins in the wild type and Fer-1-3-4 mutant after long-term exposure to excess-iron conditions. The proteins were extracted from the wild type (WT) and Fer-1-3-4 mutant, which were grown on solid half-MS agar plates supplemented with 100 mM Fe-EDTA for 10 days. The letter (c), control sample from the plants grown on only solid half-MS agar without iron supplement; the carbonylated proteins were labeled with Cy5.5-Hz. (+Fe), excess-iron treatment; the proteins were labeled with Cy7.5-Hz. (Mixed), a mixture of 5 µg of control sample labeled with Cy5.5-Hz and 5 µg of iron-treatment sample labeled with Cy7.5 hydrazide. The staining of the total proteins was carried out with Azure Red. Mr designates the protein molecular weight marker. See Figure 4b in the main text.

Supplemental Figure 7: Representative gel pictures from two additional experimental replicates of the carbonylated proteins in the wild type and Fer-1-3-4 mutant after long-term exposure to iron-deficiency conditions. The WT and ferritin triple mutant (Fer-1-3-4) plants were grown on a solid half-MS agar plate as described in Supplemental Figure 6. For iron-deficiency treatment, the medium was supplemented with 300 mM of ferrozine. Each lane contains 10 µg proteins, and the staining of the total proteins was carried out with Azure red in all the experiments. The letter (c), control sample from the plants grown on only solid half-MS agar without iron treatment; the carbonylated proteins were labeled with Cy5.5-Hz. (-Fe), iron-deficiency treatment; the proteins were labeled with Cy7.5-Hz. (Mixed), a mixture of 5 µg of control sample labeled with Cy5.5-Hz and 5 µg of iron-treatment sample labeled with Cy7.5 hydrazide. Mr designates the protein molecular weight marker. See Figure 4c in the main text.

Supplemental Figure 8: Representative pictures of the wild type (WT) and Fer-1-3-4 mutants stained for the detection of hydrogen peroxide (H2O2) and superoxide radical (O2•−) after the

iron treatments. The wild type (WT) and Fer-1-3-4 mutant exposed to excess iron (+Fe), or iron-deficiency (-Fe) conditions for 10 days were used. Histochemical detection of H2O2 and O2•− were performed using 3,3'-diaminobenzidine (DAB) and nitrotetrazolium blue chloride (NBT) staining, respectively. Seedlings were grown in half-strength MS medium without a supplement to serve as a control. The scale bar represents 1 cm.

Supplementary Figure 9: Gene ontology enrichment analysis of the carbonylated proteins identified both in the control and iron-deficiency-treated plant samples. The analysis was performed only on the carbonylated proteins identified in the three biological replicates of each treatment. (A, B) The bubble chart of the top 20 Gene Ontology (GO)-terms was generated using the ShinyGO web-based bioinformatics resource. The y-axis represents GO terms while the x-axis indicates the value of fold enrichment. (C) The subcellular localization of the carbonylated proteins was predicted by using the Subcellular Proteomic Database SUBA.

Supplemental Table S1. Primer sequences used in this study.

Supplemental Table S2. List of reactive carbonyl specie (RCS) modification of proteins. Protein carbonylation derived by an electrophilic α, β-unsaturated aldehyde produced during lipid peroxidation leaving a reactive carbonyl group on the target protein. This includes malondialdehyde (MDA),4-Hydroxy-2-nonenal (HNE), Acrolein, 4-oxo-nonenal (ONE), 4-Hydroxy-2-hexenal (HHE), 4-oxo-2-hexenal (OHE), Crotonaldehyde, Cinnamaldehyde and Pentanal

Supplementary Table S3: Full list of the carbonylated proteins identified in three biological replicates of the WT control samples only.

Supplementary Table S4: Full list of the carbonylated proteins identified in three biological replicates of the WT iron-deficient samples only

Supplementary Table S5: Full list of the carbonylated proteins identified in three biological replicates of both WT control and Fe-deficient samples.

Supplementary Table S6: UniProt identifiers of all proteins identified as carbonylated proteins using manual curation for WT control samples and Fe-deficient samples
