# Supplementary figures and images for: Iron Availability Influences Protein Carbonylation in Arabidopsis thaliana Plants
Source: Int J Mol Sci. 2023 Jun 4;24(11):9732. doi: 10.3390/ijms24119732 (PMC10253855; doi:10.3390/ijms24119732)

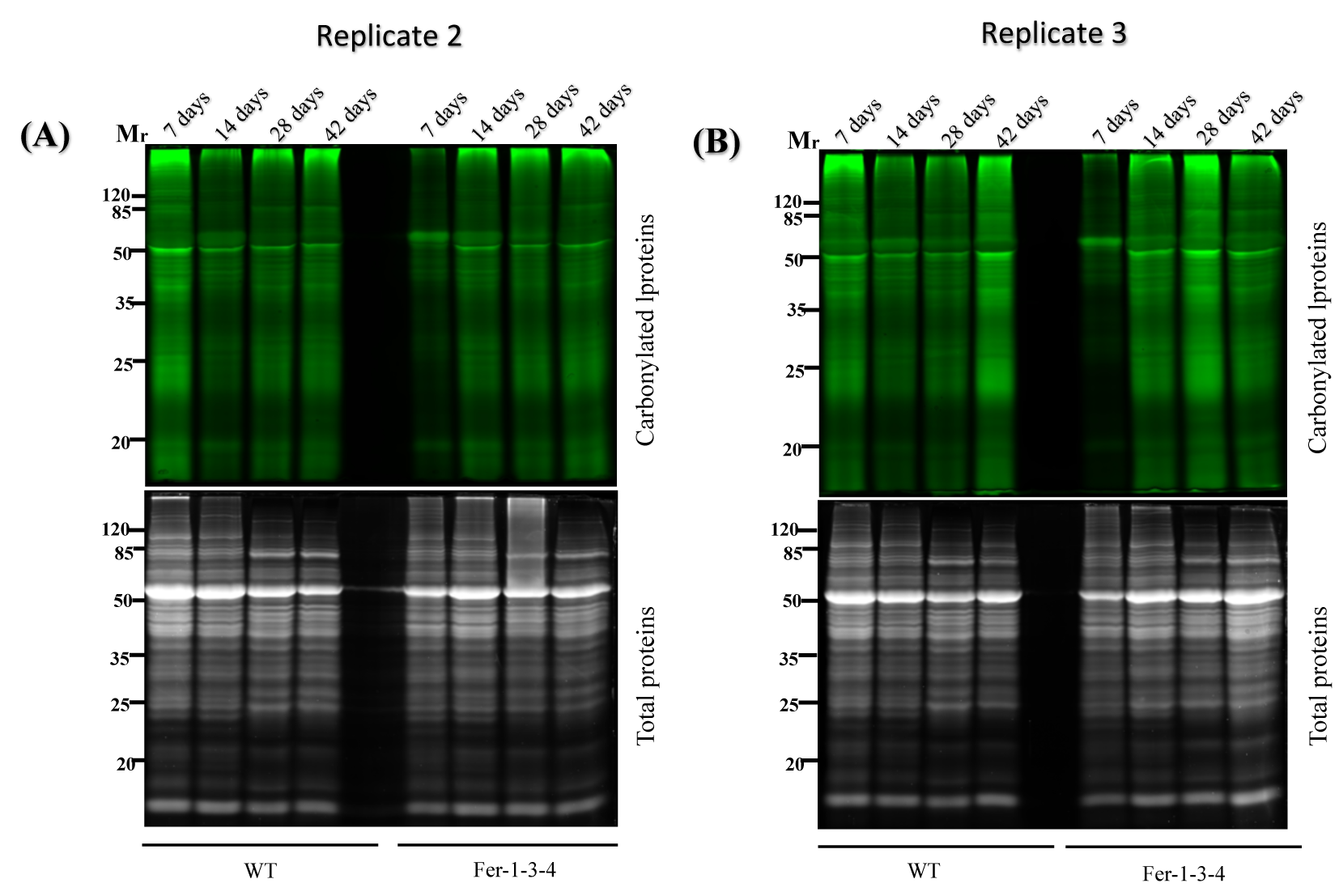

Supplement: Supplementary file 1 [file ijms-24-09732-s001.zip › Supplemental Figure 1.tif]

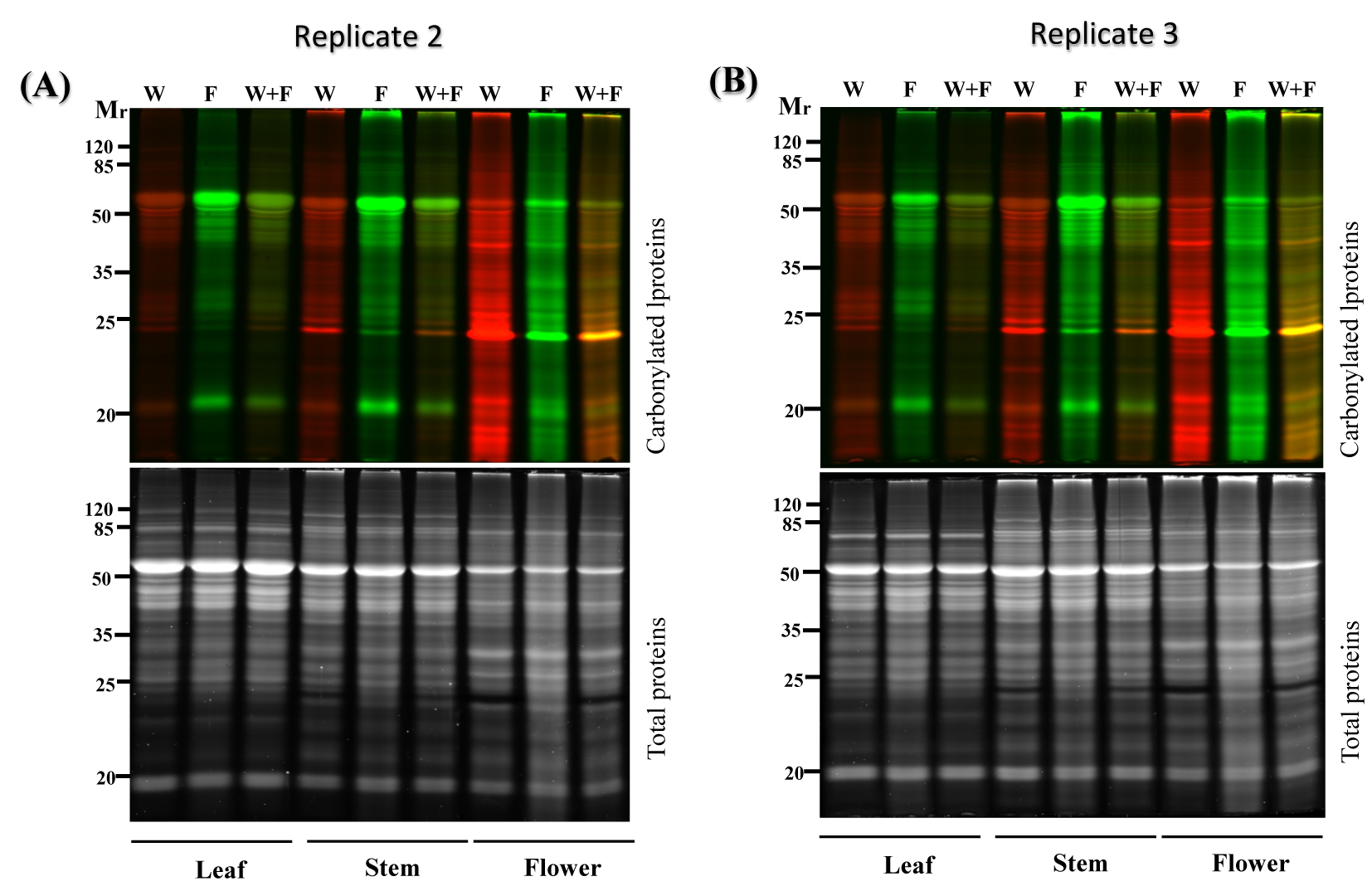

Supplement: Supplementary file 1 [file ijms-24-09732-s001.zip › Supplemental Figure 2.tif]

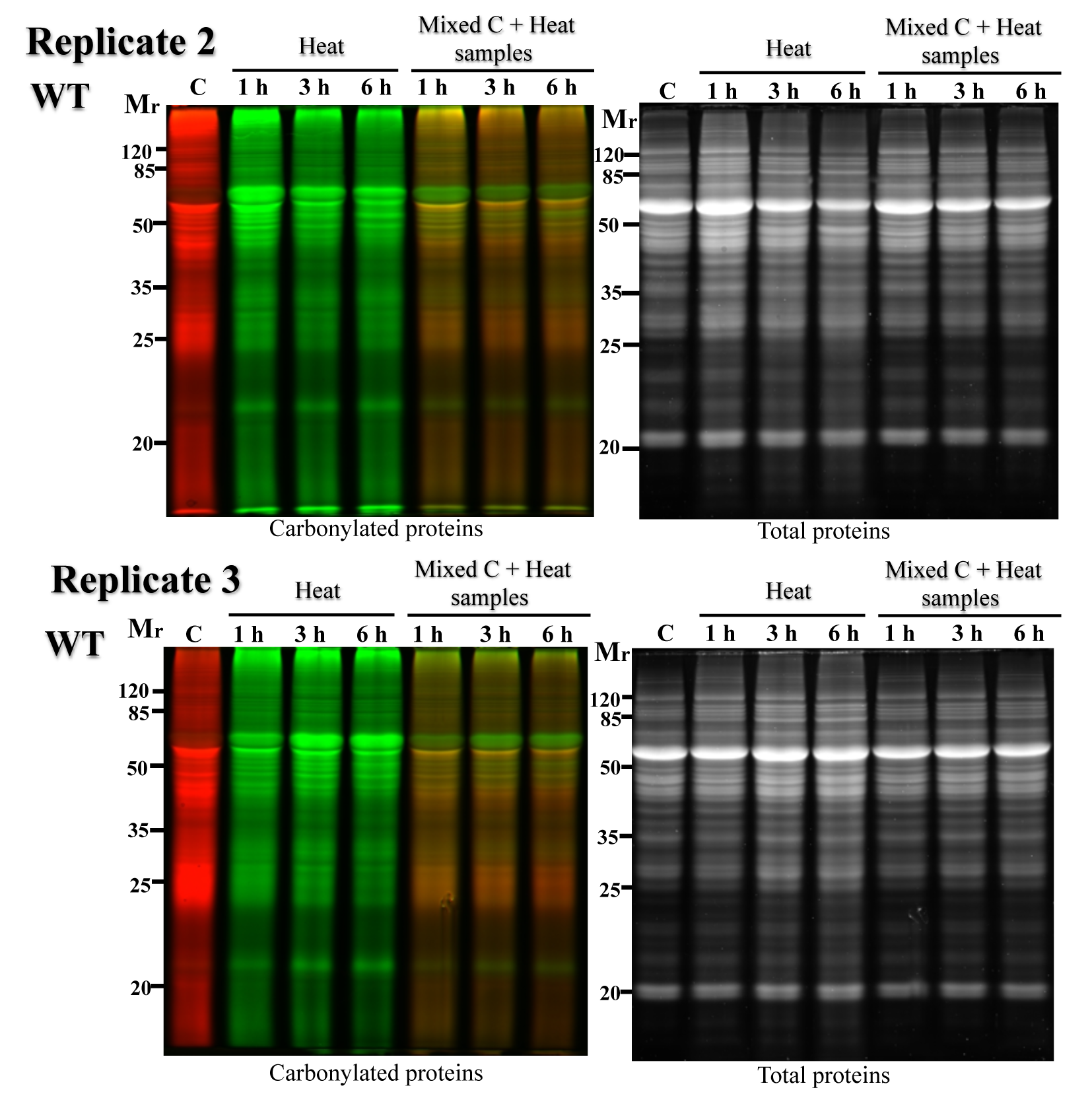

Supplement: Supplementary file 1 [file ijms-24-09732-s001.zip › Supplemental Figure 3.tif]

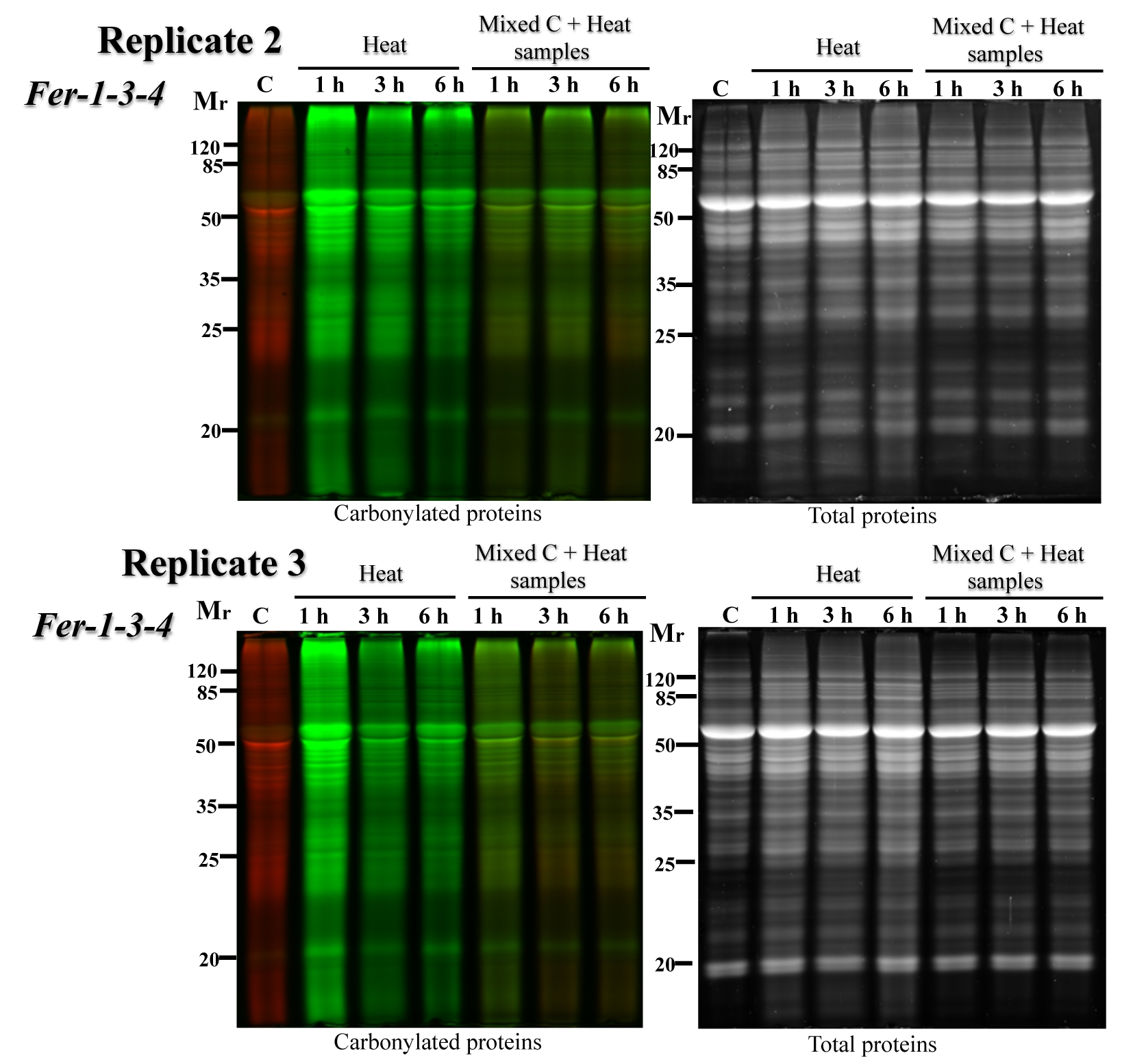

Supplement: Supplementary file 1 [file ijms-24-09732-s001.zip › Supplemental Figure 4.tif]

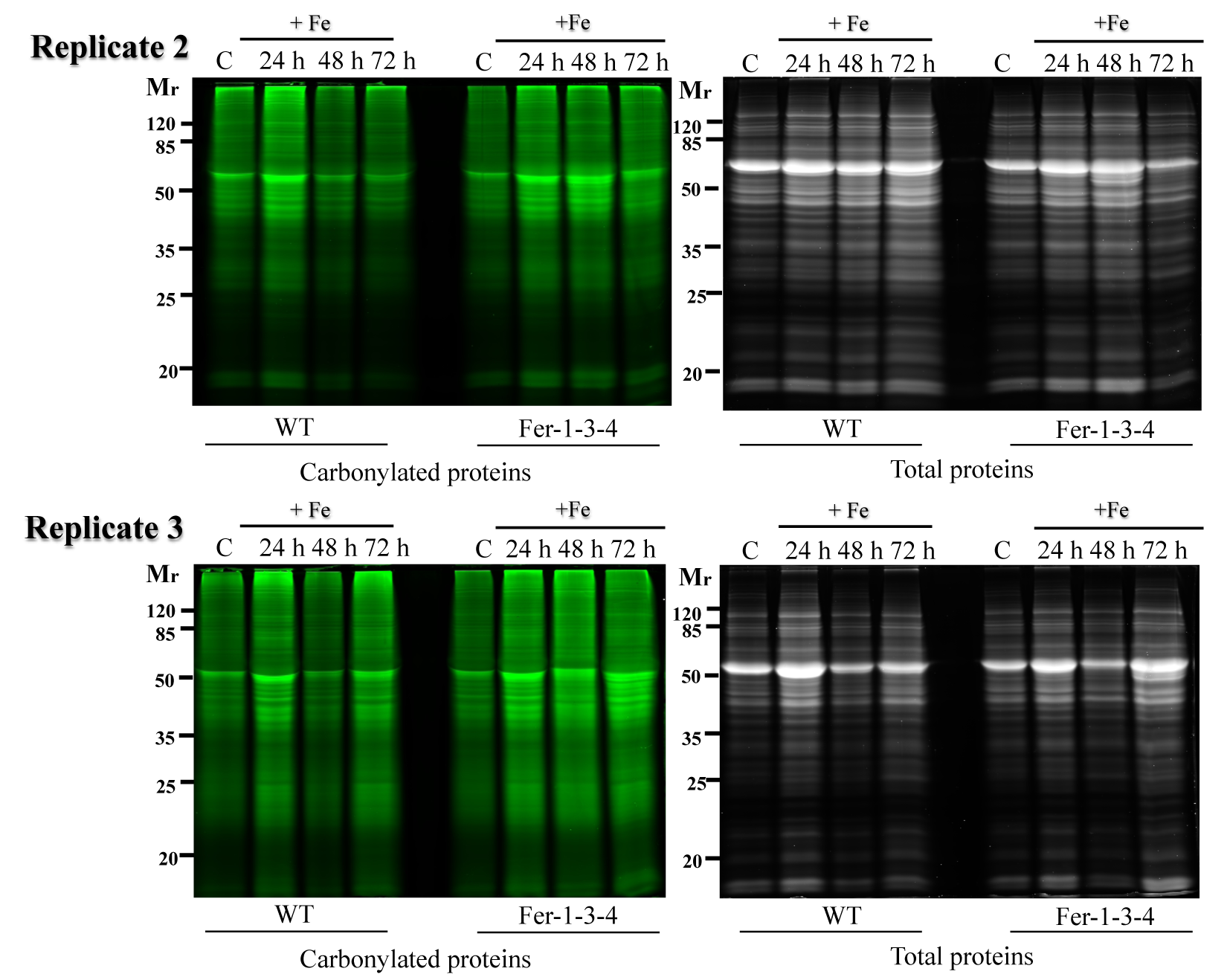

Supplement: Supplementary file 1 [file ijms-24-09732-s001.zip › Supplemental Figure 5.tif]

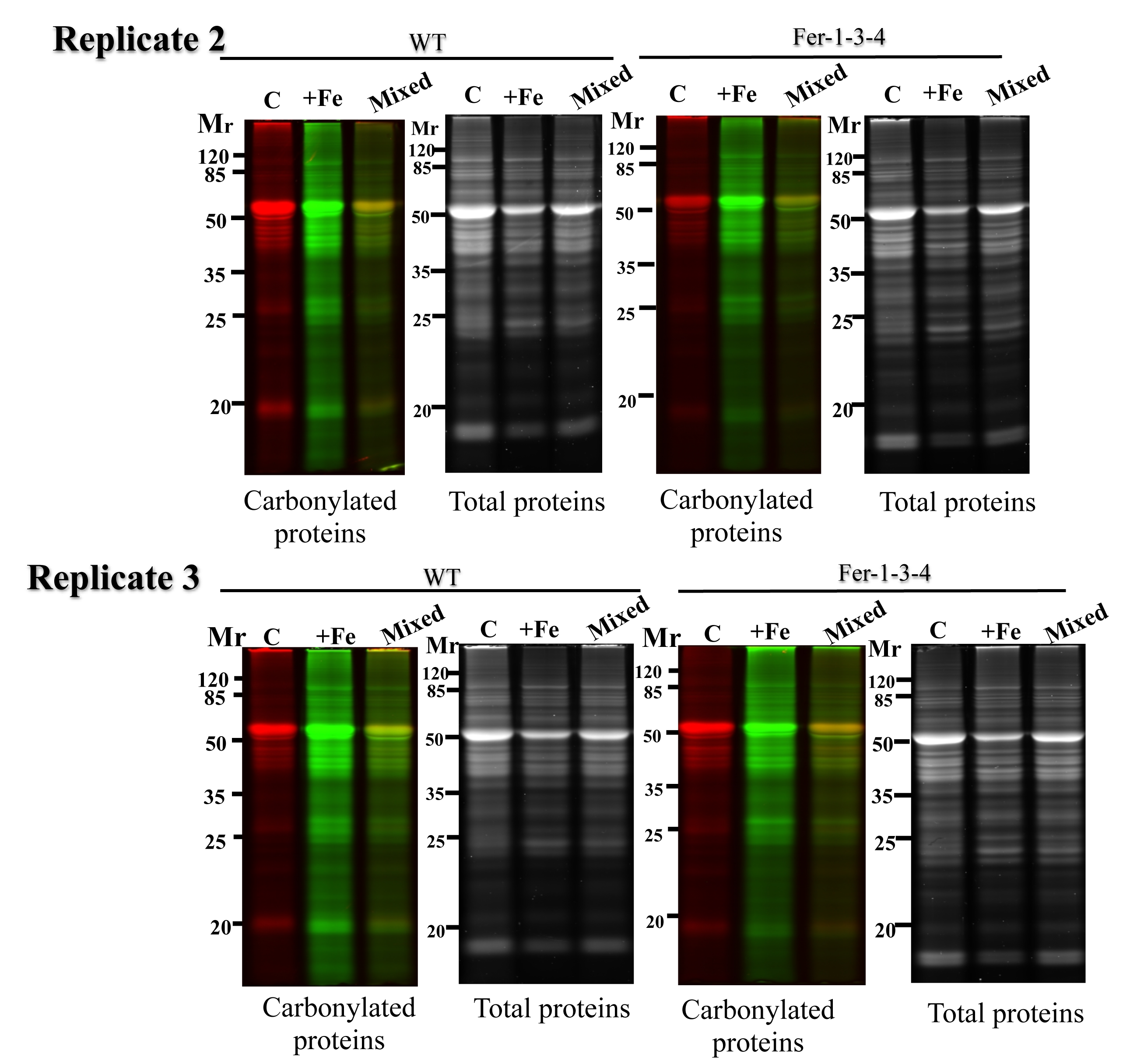

Supplement: Supplementary file 1 [file ijms-24-09732-s001.zip › Supplemental Figure 6.tif]

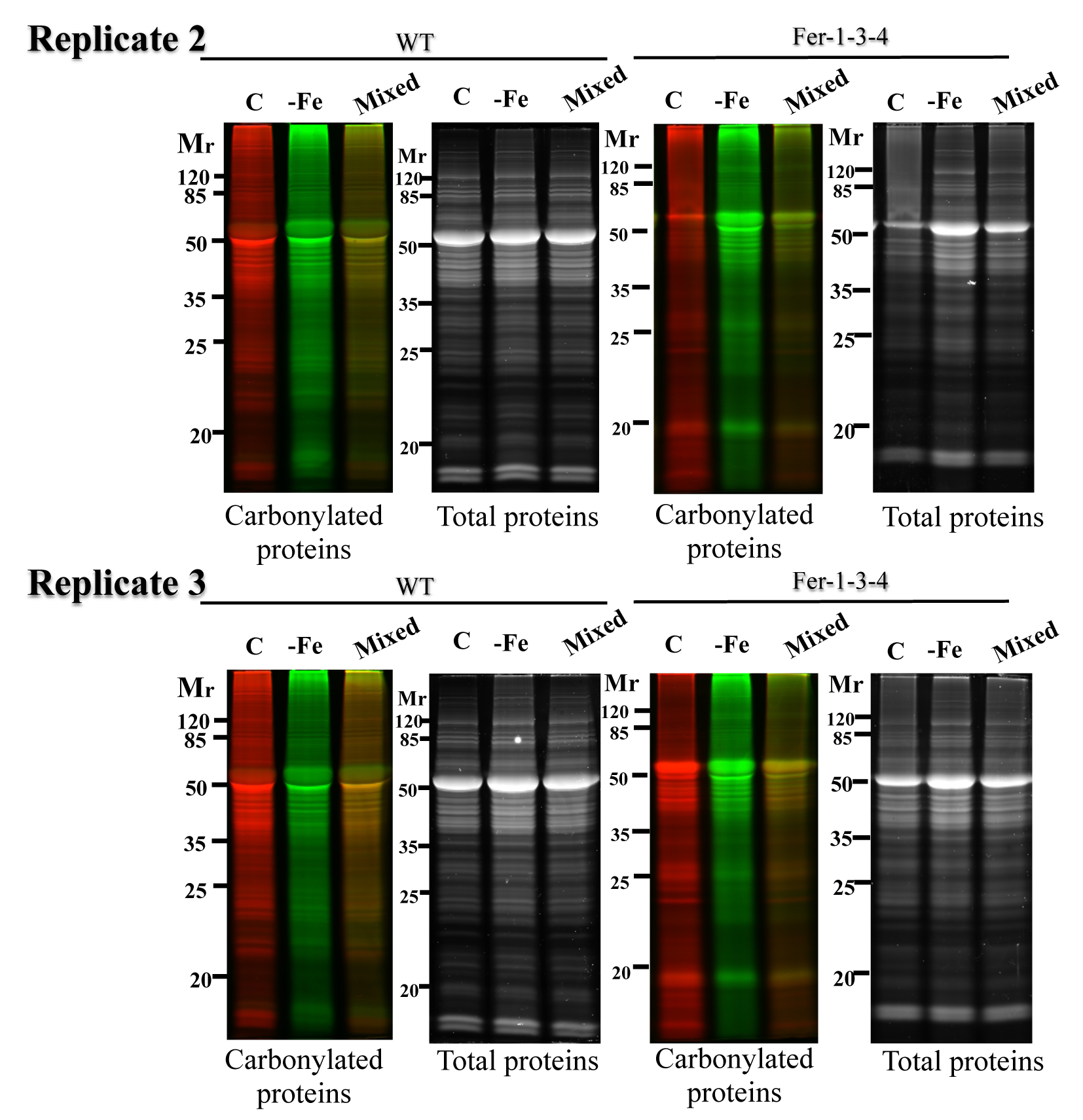

Supplement: Supplementary file 1 [file ijms-24-09732-s001.zip › Supplemental Figure 7.tif]

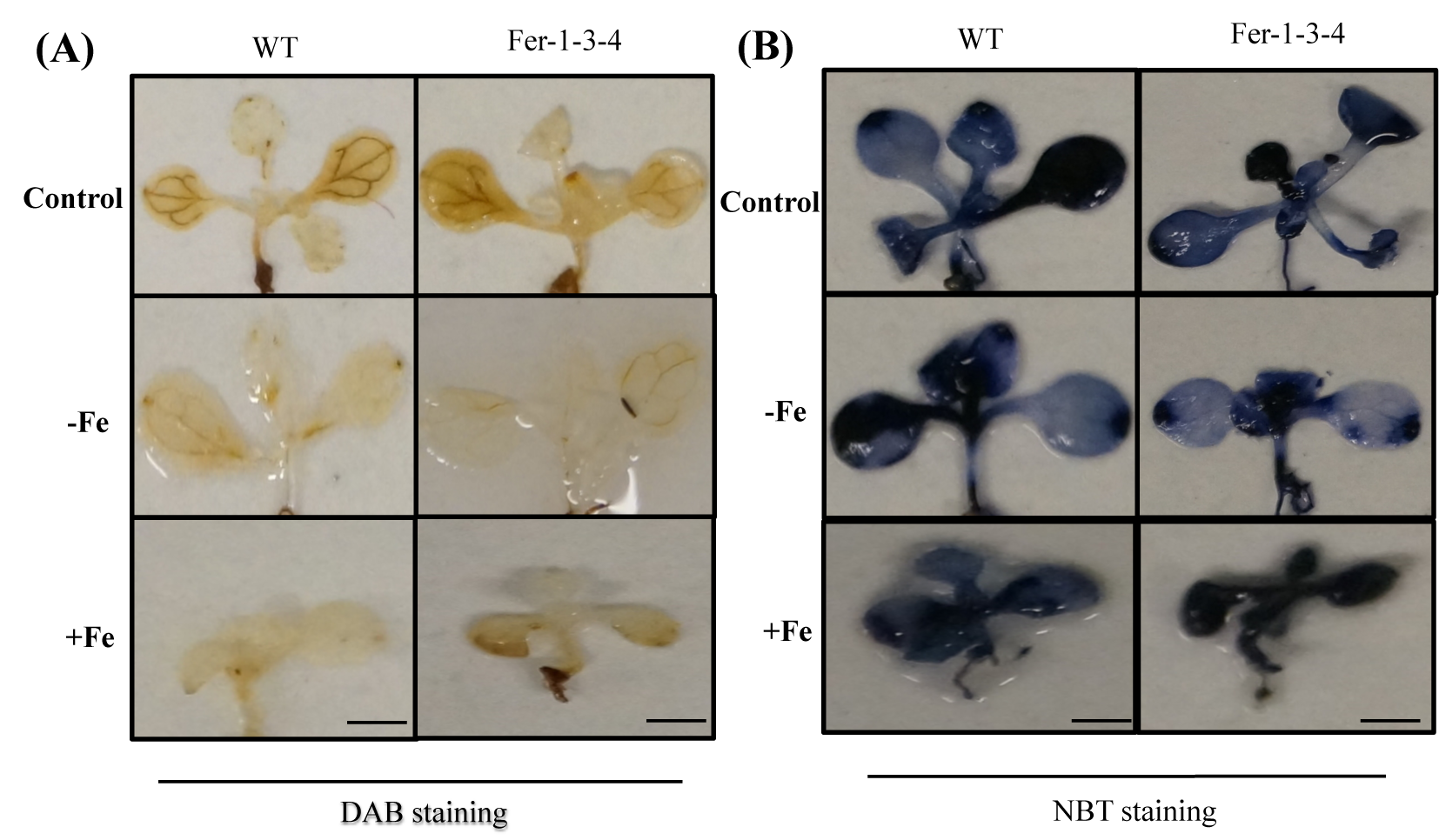

Supplement: Supplementary file 1 [file ijms-24-09732-s001.zip › Supplemental Figure 8.tif]

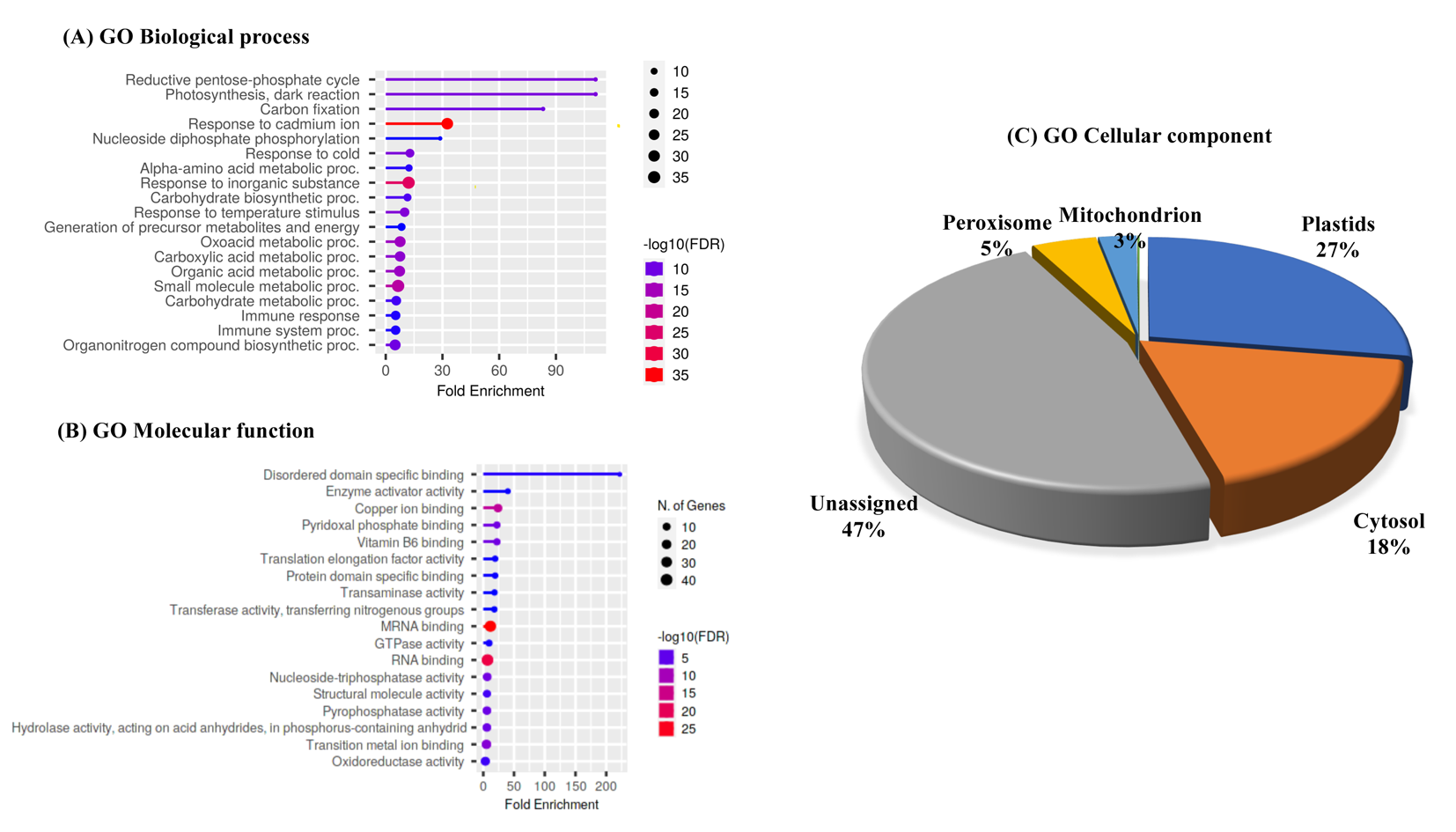

Supplement: Supplementary file 1 [file ijms-24-09732-s001.zip › Supplemental Figure 9.tif]
